# Recruitment of the Major Vault Protein by InlK: A *Listeria monocytogenes* Strategy to Avoid Autophagy

**DOI:** 10.1371/journal.ppat.1002168

**Published:** 2011-08-04

**Authors:** Laurent Dortet, Serge Mostowy, Ascel Samba Louaka, Edith Gouin, Marie-Anne Nahori, Erik A.C. Wiemer, Olivier Dussurget, Pascale Cossart

**Affiliations:** 1 Institut Pasteur, Unité des interactions Bactéries-Cellules, Paris, France; 2 INSERM, U604, Paris, France; 3 INRA, USC 2020, Paris, France; 4 Service de Bactériologie-Virologie, Hôpital de Bicêtre, Assistance Publique/Hôpitaux de Paris, Faculté de Médecine et Université Paris-Sud, Le Kremlin- Bicêtre Cedex, France; 5 Department of Medical Oncology, Erasmus University Medical Center, Rotterdam, The Netherlands; 6 Université Paris Diderot-Paris 7, Paris, France; Duke University, United States of America

## Abstract

*L. monocytogenes* is a facultative intracellular bacterium responsible for listeriosis. It is able to invade, survive and replicate in phagocytic and non-phagocytic cells. The infectious process at the cellular level has been extensively studied and many virulence factors have been identified. Yet, the role of InlK, a member of the internalin family specific to *L. monocytogenes*, remains unknown. Here, we first show using deletion analysis and *in vivo* infection, that InlK is a *bona fide* virulence factor, poorly expressed *in vitro* and well expressed *in vivo*, and that it is anchored to the bacterial surface by sortase A. We then demonstrate by a yeast two hybrid screen using InlK as a bait, validated by pulldown experiments and immunofluorescence analysis that intracytosolic bacteria *via* an interaction with the protein InlK interact with the Major Vault Protein (MVP), the main component of cytoplasmic ribonucleoproteic particules named vaults. Although vaults have been implicated in several cellular processes, their role has remained elusive. Our analysis demonstrates that MVP recruitment disguises intracytosolic bacteria from autophagic recognition, leading to an increased survival rate of InlK over-expressing bacteria compared to InlK^−^ bacteria. Together these results reveal that MVP is hijacked by *L. monocytogenes* in order to counteract the autophagy process, a finding that could have major implications in deciphering the cellular role of vault particles.

## Author Summary

*L. monocytogenes* is a food-born pathogen responsible for listeriosis, a severe illness with a high mortality rate, which mainly affects immunocompromised patients and pregnant women. The bacterium is a facultative intracellular pathogen able to invade, survive and multiply in large variety of cells. Although the infectious process at the cellular level has been extensively studied, the role of InlK, a surface protein specific to *L. monocytogenes*, remains unknown. Here we established that *L. monocytogenes* use InlK to interact with a mammalian cytoplasmic protein named Major Vault Protein (MVP). Although MVP has been implicated in diverse cellular processes, its role remains elusive. Here we demonstrate that, inside the cell, *L. monocytogenes* is able, *via* InlK, to decorate its surface with MVP to escape autophagy, an innate immune defense system that protects the cell from invading pathogens. *L. monocytogenes* uses this camouflage strategy to efficiently survive inside cells.

## Introduction

*Listeria monocytogenes* is a Gram-positive bacterium responsible for listeriosis, a severe food-borne human infection with an overall mortality rate of 30% [1]. *L. monocytogenes* has evolved efficient strategies to survive in the intestine and cross the intestinal, blood-brain and placental barriers [2], [3] leading to clinical features of the disease that include gastroenteritis, septicemia, central nervous system infections, and mother-to-child infections [4]. Inside the host, this facultative intracellular bacterium is able to invade phagocytic and non-phagocytic cells, replicate intracellularly, and spread directly from cell-to-cell, thereby escaping the immune response [3]. *L. monocytogenes* has thus emerged as a paradigm to study host-pathogen interactions and fundamental processes in cell biology [5]. For instance, the study of actin rearrangements upon entry and intracellular movements [6]–[9] is an example of how understanding a bacterial-induced process can yield insight into basic cellular processes. Namely, the listerial virulence factor ActA triggers the recruitment of Arp2/3 complex and Ena/VASP to mediate actin polymerization and propel the bacterium from one infected cell to another without exposure to the extra-cellular milieu [8], [10]. Interestingly, as shown recently ActA also disguises the bacteria from autophagic recognition within the cytosol as ActA- bacteria becomes rapidly ubiquitinated and targeted to autophagy [9], [11]. It is currently viewed that ubiquitin-associated bacteria recognized by the autophagy machinery are trapped by autophagosomal membrane for delivery into the lytic compartment where they undergo degradation by autolysosomes [11], [12]. Interestingly, a variety of studies had noticed that autophagic markers can accumulate around intracytosolic *L. monocytogenes*, unless bacteria were forming actin tails [13], [14]. Consequently, it has been hypothesized and shown that *L. monocytogenes* avoids ubiquitination and autophagic recognition by expressing ActA, and ActA mutants are efficiently targeted by autophagy [11]. While the role of ActA in autophagy is now established, the role that many other surface proteins play during *Listeria* infection remains fragmentary [15].

The vault particle is the largest cytoplasmic ribonucleoprotein complex known to date [16]. Originally identified as contaminants of clathrin-coated vesicles preparation, these complexes were named vault particles because of their barrel shaped morphology resembling the ceiling of cathedrals [17]. Mammalian vaults are composed of the highly conserved major vault protein (MVP) constituting more than 70% of the mass of the particle [16], [18], [19] which spontaneously forms vault particles without the need of other vault components [20]. The two other vault components are the telomerase associated protein (TEP-1) [21] and the vault poly(ADP)ribose polymerase (vPARP) [22]–[24]. Vault preparations have additionally been shown to contain several small untranslated RNAs [25], [26]. Vaults exist in thousands of copies per cell and are widely expressed in all eukaryotic organisms, from *Dictyostelium discoideum* to mammals, except plants, *Saccharomyces cerevisiae*, *Caenorhabditis elegans* and *Drosophila melanogaster* [27]. Diverse roles have been proposed for MVP and/or vaults [27], including roles in drug resistance [28], cellular differentiation [29], innate immunity [30], virus infections [31], signaling cascades [28], [32]–[35] and cell survival [33], [36]. However, the precise cellular function(s) of MVP and vaults remains poorly understood. In addition, the *MVP* ^−/−^ mice are viable, healthy and show no obvious abnormalities [37], [38].

The genome sequence of *L. monocytogenes* EGD-e has revealed the presence of 25 genes encoding proteins of the internalin family [39]. Proteins of this family, which are characterized by the presence of leucine-rich-repeats (LRRs), are mostly surface proteins [40]. Their binding to the bacterial surface is mediated by different anchoring domains, in particular the LPXTG motif which allows a sortase A mediated covalent attachment to the peptidoglycan [41]. The invasion protein, Internalin, is one such protein [42]. Comparative post-genomic studies have established that several members of the *L. monocytogenes* internalin family are absent in *L. innocua*, a closely related non-pathogenic species [40]. *Lmo1290* is an internalin gene absent in *L. innocua*, herein referred to as *inlK*, which is expressed at very low levels in brain-heart-infusion medium [43], [44] and induced during infection [43].

In this study we investigated the role of InlK in the infectious process. We first explored the expression of InlK and the virulence phenotype of the *inlK* deletion mutant. We then searched for potential host partners of InlK and identified MVP. We demonstrated that the InlK/MVP interaction occurs in the cytosol of infected cells at the bacterial surface. Moreover, our results reveal that MVP recruitment protects *L. monocytogenes* from autophagic recognition, leading to an increase in bacterial survival in infected cells.

## Results

### *L. monocytogenes inlK* encodes a virulence factor

**Figure 1:**
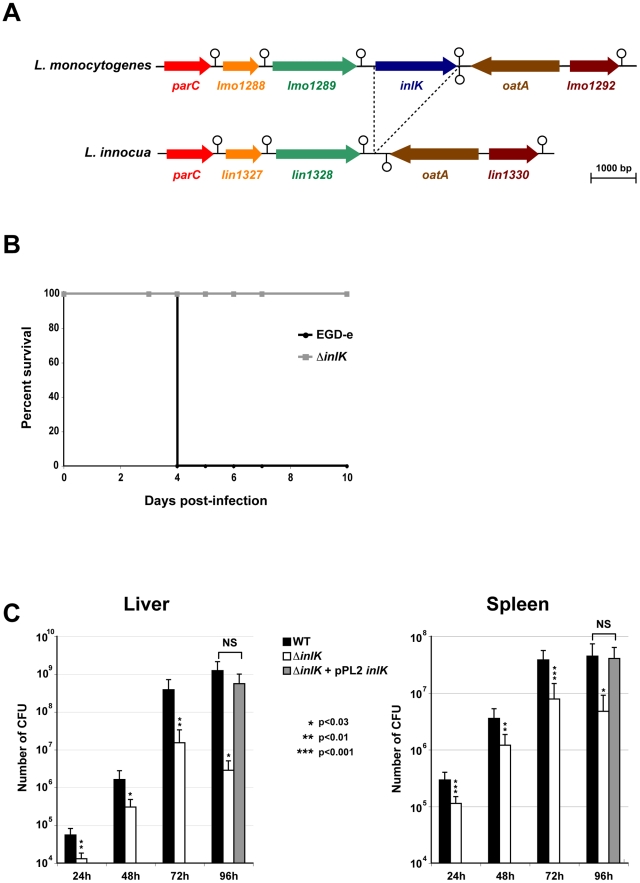
InlK is a virulence factor of *L.* monocytogenes. A. The *inlK* gene locus in *L. monocytogenes* compared with the same genomic region in the related non-pathogenic species *L. innocua*. The stem and circle represent transcription terminators. B. Kaplan-Meier curve represents the survival of BALB/c mice over time**.** Four BALB/c mice in each experimental group were infected i.v with 10^4^ *L. monocytogenes* wild-type (EGD-e) or Δ*inlK* mutant. C. The *L. monocytogenes* EGD-e wild-type strain (WT), the Δ*inlK* mutant (Δ*inlK*) and the complemented strain (Δ*inlK*+pPL2 *inlK*) 10^4^ CFU were inoculated i.v into BALB/c mice. Animals were euthanized 24 h, 48 h, 72 h or 96 h after infection and organs were recovered, homogenized, and homogenates serially plated on BHI. The number of bacteria able to colonize liver (left panel) and spleen (right panel) is expressed as log_10_ CFU. Four animals per bacterial strain, per time points and per experiment were used. Statistical analyses were performed on the results of 3 independent experiments using the Student *t* test. *P* values of <0.05 were considered statistically different and are labeled here as *.

The gene *lmo1290* ( = *inlK*) is 1797 bp long. It is located 331 bp downstream from gene *lmo1289* which is followed by a transcriptional terminator. *Lmo1290* is also followed by a transcriptional terminator upstream from the divergently transcribed *oatA* gene which encodes a peptidoglycan O–acetyltransferase (Figure 1A) [45]. The *inlK* gene is present in all 22 *L. monocytogenes* genomes sequenced to date and absent from the genomes of *L. ivanovii* and all non-pathogenic *Listeria* strains including *L. innocua* (Figure 1A), *L. seeligeri*, *L. welschimeri* and *L. grayi*, suggesting that InlK could be involved in *Listeria* virulence.

To assess the role of InlK in virulence, we generated an *inlK*-deletion mutant (Δ*inlK*) in the strain EGD-e. The Δ*inlK* mutant grew as rapidly as the wild-type (WT) in broth medium and infected cells (macrophages and epithelial HeLa cells) (data not shown). The LD50 of the Δ*inlK* mutant after intravenous (i.v.) injection in BALB/c mice was 2.2×10^4^ CFU, compared with 1.7×10^3^ CFU for the WT strain. Inactivation of *inlK* resulted in complete survival of animals infected intravenously with 10^4^ bacteria (Figure 1B). In contrast, infection with the same number of WT bacteria led to 100% mortality. Moreover, the number of CFU recovered from spleens and livers of i.v. infected BALB/c mice after 24 h, 48 h, 72 h and 96 h of infection was significantly lower (∼1 Log_10_) for the mutant compared to the WT (Figure 1C), and virulence of the mutant was fully restored by complementation (Figure 1C). Together, these results establish a role for InlK in the virulence of *L. monocytogenes*.

### InlK is expressed *in vivo*

**Figure 2:**
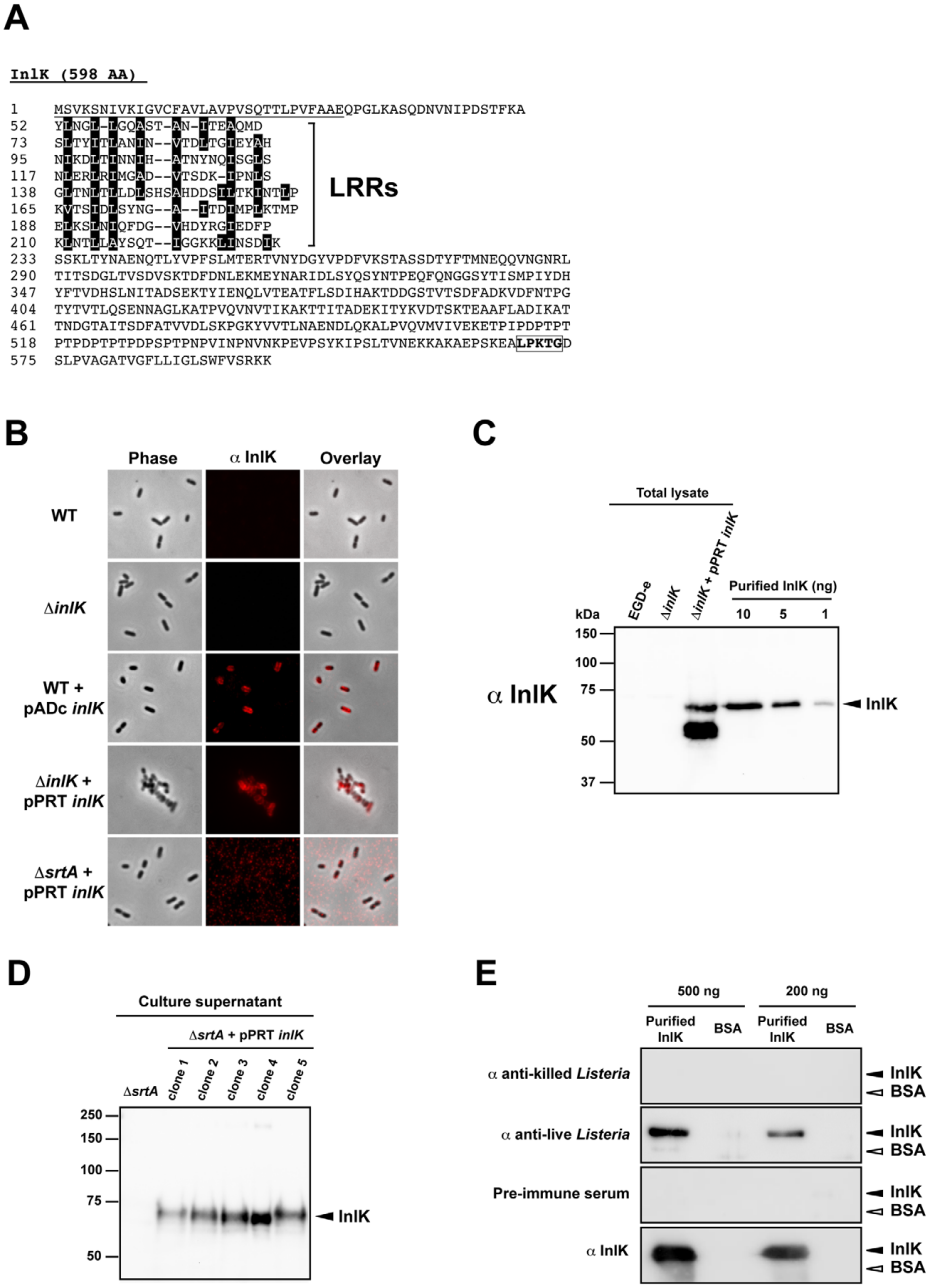
InlK is expressed *in vivo*. A. InlK amino acid sequence. The signal sequence is underlined and the different regions of leucine rich repeats (LRRs) are outlined. The consensus pentapeptide LPXTG at the C-terminal end is boxed. B. Detection by immunofluorescence microscopy of InlK over-expressing in *L. monocytogenes* EGD-e (WT), Δ*inlK,* WT+pADc*-inlK,* Δ*inlK+*pPRT*-inlK* and the Δ*srtA* mutant over-expressing inlK (Δ*srtA*+pPRT*-inlK*) grown in BHI medium using the rabbit polyclonal anti-InlK antibody. InlK was detected at the surface of InlK over-expressing bacteria (WT+pADc*-inlK* and Δ*inlK+*pPRT*-inlK*), whereas it was undetectable at the surface WT bacteria or at the surface of the Δ*strA* mutant over-expressing inlK. C. Detection of InlK by Western blot on total lysates of *L. monocytogenes* EGD-e (WT), Δ*inlK* and Δ*inlK+*pPRT*-inlK* grown in BHI using the rabbit polyclonal anti-InlK antibody. Decreased concentrations of recombinant purified InlK were used as a positive control. D. Detection of secreted InlK in the supernatant of Δ*srtA* mutants over-expressing InlK. Western blotting was carried out on trichloroacetic acid precipitates of Δ*srtA* and Δ*srtA*+pPRT-*inlK* culture (OD_600_ = 1) supernatants using the rabbit polyclonal anti-InlK antibody. E. Detection of purified recombinant InlK protein with rabbit polyclonal anti-live *Listeria* antibody, rabbit polyclonal anti-killed *Listeria* antibody, rabbit polyclonal anti-InlK and a rabbit pre-immune serum. InlK was detected only with the rabbit polyclonal anti-live *Listeria* antibody indicating that it is expressed during the *in vivo* infectious process. BSA was used as control protein. Two different amounts of proteins were tested (500 ng and 200 ng) to access signal specificity.

InlK is a 598 amino acid LPXTG surface protein predicted to be anchored to the peptidoglycan by sortase A (Figure 2A). To address whether *L. monocytogenes* produces InlK *in vitro*, we first generated an antibody against a purified recombinant InlK protein (Figure S1A) and used it to detect the protein at the bacterial surface by immunofluorescence. In agreement with previous whole genome transcriptomic results that demonstrated a low expression level of *inlK in vitro* [44], bacteria grown in brain-heart infusion (BHI) medium were not stained by the InlK antibody (Figure 2B), suggesting that InlK protein was poorly expressed on the surface or not produced. We then showed that InlK was not detected in bacterial total extracts (Figure 2C), also in agreement with previous data indicating that InlK is not present in the cell wall proteome of *L. monocytogenes* EGD-e grown in BHI medium [46]. Moreover, consistent with the fact that the two major regulators of virulence genes, PrfA and sigmaB, were not required for basal *inlK* transcription [44], [47], the InlK protein level was also not detectable when bacteria were grown in charcoal supplemented medium or at low pH (data not shown).

To verify that the *inlK* open reading frame encoded a surface protein, *inlK* was expressed under the control of two constitutive promoters active in *Listeria*. We used either the promoter of the protease gene from *Lactococcus lactis* subsp. *cremoris* on the multicopy plasmid pPRT-*inlK* or the promoter P_Hyper_ after integration on the chromosome of the plasmid pADc*-inlK* [48], [49]. InlK antibodies efficiently labeled InlK on the surface of bacteria that constitutively expressed *inlK* (Figure 2B) and also detected the protein in bacterial total extracts (Figure 2C). This labeling was specific, as the InlK antibody did not label WT or *inlK* mutant bacteria grown in same conditions. Interestingly, when InlK was over-expressed by *Listeria* under the control of constitutive promoters, a polypeptide with a lower mass than expected was also detected by Western-blot (Figure 2C) indicating that the protein may be processed. Moreover, InlK was not detected by immunofluorescence at the surface of a Δ*srtA* sortase mutant over-expressing *inlK* (Figure 2B), but was then detected in the supernatant of the culture medium (Figure 2D). Taken together these results established that, when *inlK* is expressed, the protein is anchored at the bacterial surface in a sortase A-dependent manner.

Recently, a whole genome transcriptomic analysis of *L. monocytogenes* during infection revealed that the gene *inlK* was better expressed *in vivo* compared to growth in BHI [43]. We thus investigated whether the InlK protein was indeed produced *in vivo* by testing for the presence of anti-InlK antibodies. Purified InlK was submitted to migration on polyacrylamide gel (Figure S1A) and blotted with two different rabbit anti-*Listeria* sera. As shown in Figure 2E, a rabbit anti-*L. monocytogenes* serum obtained after immunization with killed bacteria was not able to detect the purified InlK whereas the serum directed against live bacteria detected InlK. This signal was specific to InlK as the antibodies did not label bovine serum albumine (BSA) used at the same concentration.

To confirm *in vivo inlK* expression, we constructed an expression reporter vector in which the expression of the bioluminescent operon *lux*_ABCDE_ was under the control of *inlK* promoter (pPL2-P*_inlK_*-*lux*_ABCDE_). This construct was integrated in the chromosome of WT *L. monocytogenes* EGD-e, and the resulting strain was used to infect cell lines or BALB/c mice [50]. As shown in Figure S1B, *inlK* was neither expressed in BHI growth medium (right panel) nor in cells infected with bacteria previously grown in BHI (left panel). Conversely, *inlK* was expressed *in vivo* in i.v. infected mice, 24 h post-infection (Figure S1C). This signal was specific to *inlK* expression as it did not superimpose on those obtained with the control strain of *L. monocytogenes* that contains a bioluminescent reporter of LLO promoter (pPL2-P*_hly_*-*lux*_ABCDE_). Together, these results confirm that InlK is expressed *in vivo*.

### InlK interacts with the Major Vault Protein

**Figure 3:**
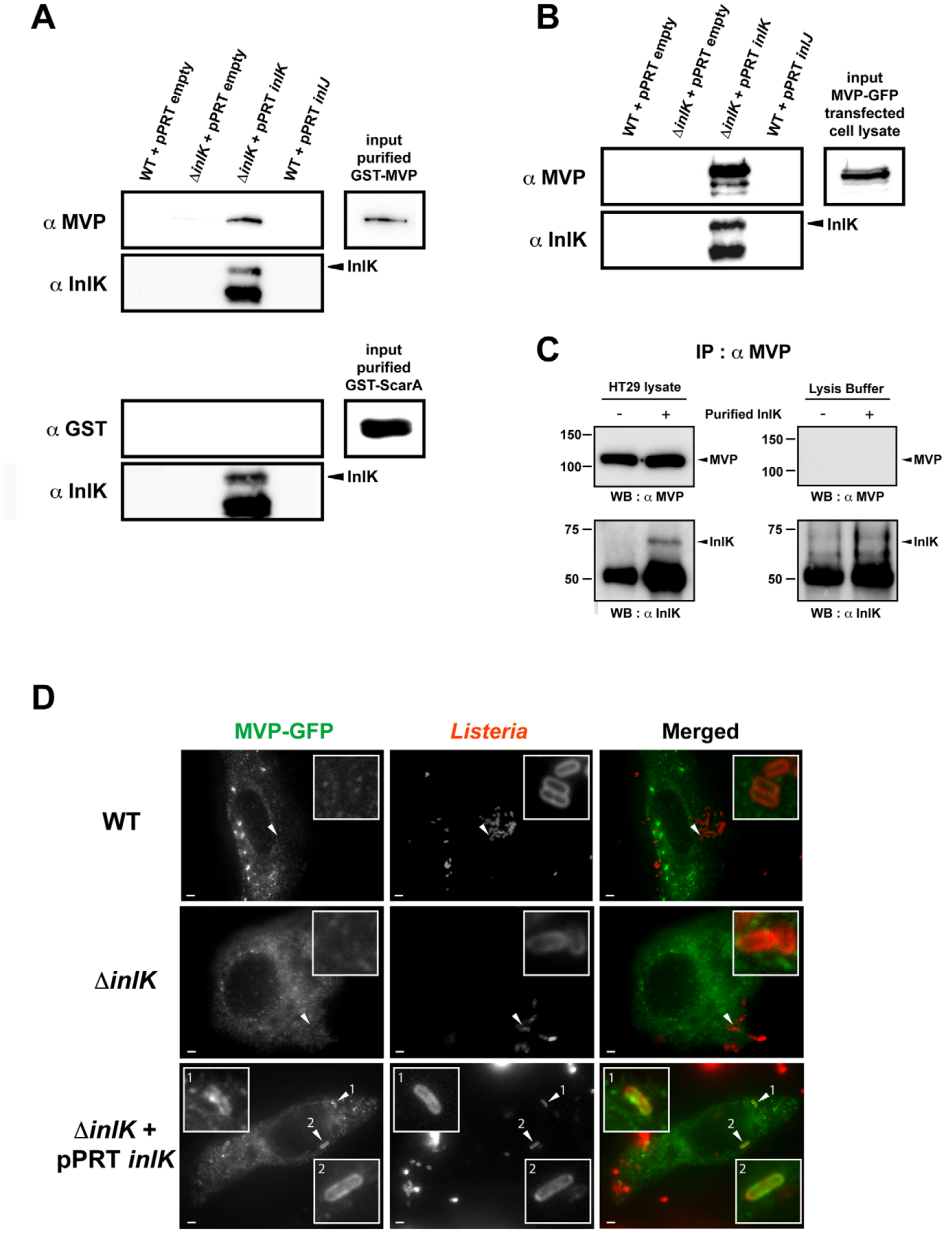
InlK interacts with the Major Vault Protein. A. Bacterial pull-down of purified GST-MVP with the *L. monocytogenes* strains WT+pPRT-empty, Δ*inlK*+pPRT-empty, Δ*inlK*+pPRT-*inlK* and WT+pPRT-*inlJ*. GST-MVP bound to InlK over-expressing bacteria (Δ*inlK*+pPRT-*inlK*) but not to other bacteria. GST-ScarA was used as control of the specificity of the MVP precipitation by InlK over-expressing bacteria. B. Bacterial pull-down of MVP-GFP from transfected HeLa cell lysates with the *L. monocytogenes* strains WT+pPRT-empty, Δ*inlK*+pPRT-empty, Δ*inlK*+pPRT-*inlK* and WT+pPRT-*inlJ*. MVP-GFP bound to InlK over-expressing bacteria (Δ*inlK*+pPRT-*inlK*) but not to other bacteria. C. Co-immunoprecipitation (Co-IP) of purified InlK (20 µg) with endogenous MVP of HT29 cells. The right panel shows anti-MVP co-IP performed on HT29 cell lysates. The left panel shows the control anti-MVP co-IP performed on lysis buffer. D. Detection of MVP recruitment at the surface of InlK over-expressing bacteria. HeLa cells were transfected with MVP-GFP (green), infected with *L. monocytogenes* EGD-e wild-type (WT), Δ*inlK* or Δ*inlK*+pPRT-*inlK* for 4 h, fixed for fluorescence light microscopy, and stained with anti-*Listeria* antibodies (red). Inset regions are magnified. The scale bar represents 1 µm.

To identify InlK interaction partners in the eukaryotic cell, we used InlK as a bait in a large-scale yeast two-hybrid screen and identified the Major Vault Protein (MVP) as a prey with a very high interaction score (Table S1). To confirm this interaction we performed a bacterial pull down assay and showed that GST-MVP purified protein bound to InlK over-expressing bacteria, but not to WT bacteria (Figure 3A). This interaction was specific as (i) the WT strain (which expresses InlA, InlB and InlH) did not bind MVP (data not shown), (ii) the overexpression of InlJ (i.e. another internalin not expressed in BHI [49]) was not able to mediate bacterial binding to MVP, and (iii) InlK over-expressing bacteria were not able to bind another GST fusion protein, GST-ScarA. Finally, bacterial incubation with MVP-GFP transfected cells lysates confirmed the interaction between InlK and MVP (Figure 3B). This interaction occurred when InlK was either expressed on a multicopy plasmid, or integrated in the chromosome (Figure S2A).

This interaction between purified InlK and endogenous MVP was confirmed by co-immunoprecipitation assays (Figure 3C). Indeed, when purified InlK was incubated with HT29 cell lysate, it interacted with endogenous MVP and the two partners co-immunoprecipitated, as shown using an anti-MVP antibody (Figure 3C). Similar results were obtained with stable HEK293-HTP-InlK cells that were engineered to express InlK in their cytosol, under the control of a tetracyclin inducible promoter (Figure S2B).

### The InlK/MVP interaction occurs in the cytosol at the bacterial surface and does not depend on actin polymerization

**Figure 4:**
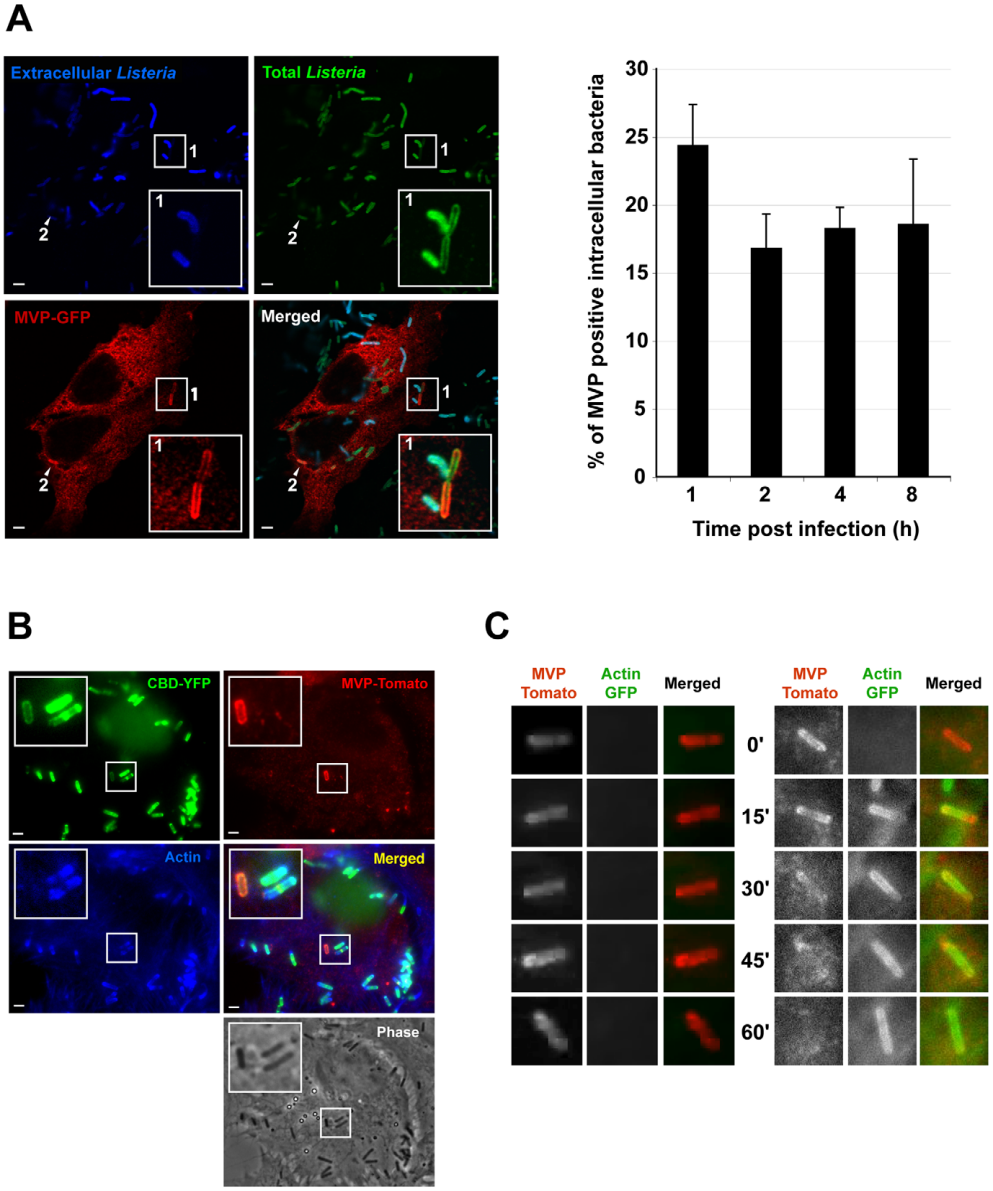
InlK/MVP interaction occurs in the cytosol, before actin polymerization. A. Detection of MVP recruitment at the surface of intracellular InlK over-expressing bacteria. HeLa cells were transfected with MVP-GFP (red), infected with InlK expressing *Listeria* (Δ*inlK*+pPRT-*inlK*) for 4 h, fixed for fluorescence light microscopy. Intra- (only green) and extracellular (cyan = green+blue) bacteria were differentially stained with anti- *Listeria* antibody (cf Material and Methods). Inset regions are magnified. Arrows indicate another intracellular bacterium which recruit MVP-GFP. The scale bar represents 1 µm. The right panel represents the quantification of the intracellular bacteria that recruit MVP (mean%±SEM%) shown in the left panel. Statistical analyses were performed on the results of 3 independent experiments using the Student's *t* test. No significant difference was found between the 4 time points. B. Detection of MVP recruitment at the surface of intracytosolic InlK over-expressing bacteria. HeLa cells were transfected with MVP-tomato (red) and YFP-CBD (green), infected with InlK over-expressing *Listeria* (Δ*inlK*+pPRT-*inlK*) for 4 h, fixed for fluorescence light microscopy and stained with phalloidin (blue). MVP positive bacteria were also labeled with YFP-CBD revealing that MVP was recruited by intracytosolic bacteria after the lysis of the internalization vacuole. Inset regions are magnified. The scale bar represents 1 µm. C. Kinetics of MVP and actin recruitment at the surface of InlK over-expressing bacteria. HeLa cells were transfected with MVP-tomato (red) and actin-GFP (green), infected with InlK over-expressing *Listeria* (Δ*inlK*+pPRT-*inlK*) for 4 h, and prepared for real-time video microscopy. Image series were collected every 15 min for 2 h. The left part shows an MVP positive bacterium that never recruits actin. The right part shows MVP replacement by actin around the bacterium. No colocalization of MVP-Tomato and actin-GFP was detected. Time is indicated along the Y axis. The entire image sequence can be viewed as Video S1.

In agreement with a specific interaction between InlK and MVP, we observed that InlK over-expressing bacteria co-localized with MVP in MVP-GFP transfected HeLa cells whereas the *inlK* mutant or wild type bacteria that do not express InlK *in vitro* did not co-localize with MVP (Figure 3D). As MVP has been mainly described as a cytoplasmic protein [51], [52] and InlK is targeted and anchored to the bacterial surface (Figure 2D), we hypothesized that the InlK/MVP interaction should occur in the cytosol of infected cells after lysis of the internalization vacuole. To test this hypothesis, we analyzed the localization of MVP recruiting bacteria. A differential immuno-staining protocol allowing extra- and intracellular *Listeria* to be distinguished showed that MVP was recruited to intracellular bacteria (Figure 4A). Irrespective of the time post-infection, ∼20% of InlK over-expressing bacteria were observed to recruit MVP [24.3%±3.0; 16.8±2.5; 18.2±1.6 and 18.5%±4.8 (mean ± SEM from n = 3 experiments) at 1 h, 2 h, 4 h and 8 h post-infection respectively] (Figure 4A, right panel). To determine whether MVP was recruited to intracellular bacteria before or after lysis of the internalization vacuole, we used a marker of early times points after vacuole escape, YFP-CBD, a YFP fused phage protein known to bind *L. monocytogenes* peptidoglycan as soon as the vacuole membrane begins to lyse (Figure S3A) [53]. Cells were co-transfected with MVP-Tomato and YFP-CBD, fixed 4 h post-infection and immuno-stained for actin. As expected, bacteria that polymerized actin were efficiently labeled with YFP-CBD (Figure 4B, Figure S3B inset 2), confirming that YFP-CBD efficiently labels intracytosolic *Listeria*. Moreover, all MVP-positive bacteria were also labeled with YFP-CBD (Figure 4B, Figure S3B) revealing that MVP was recruited by intracytosolic bacteria after lysis of the internalization vacuole.

Interestingly, we did not observe the co-recruitment of MVP-GFP and endogenous actin to intracellular bacteria (Figure 4B, Figure S3B). Co-recruitment was also not observed in infected cells previously co-transfected with MVP-GFP and actin-CFP (Figure S3C). We therefore analysed the kinetics of MVP and actin recruitment by performing live-cell imaging. Cells were co-transfected with MVP-Tomato and actin-GFP, and infected with InlK expressing *L. monocytogenes*. Strikingly, MVP was recruited rapidly by InlK over-expressing bacteria and could then be replaced by actin (Figure 4C and Video S1), showing that MVP recruitment occurs before actin polymerization. We then verified that MVP recruitment occurred independently of actin polymerization using a *actA* mutant. Intracytosolic Δ*actA* over-expressing InlK were efficiently labeled with MVP (Figure S3D). This MVP recruitment was more efficient than for wild type bacteria. Indeed, the percentage of intracytosolic Δ*actA* over-expressing InlK having recruited MVP at 4 h post-infection was 88.3±12.7% (mean ± SEM from n = 3 experiments), compared to the 16.8±2.5% (mean ± SEM from n = 3 experiments) observed when using the InlK over-expressing strains that are able to polymerize actin *via* ActA. Together, these results suggested that ActA at least partially impairs MVP recruitment. As ActA protects bacteria from autophagy [11], these data also suggested that both InlK and ActA may protect bacteria from autophagy.

### MVP recruitment protects *Listeria* from autophagy

**Figure 5:**
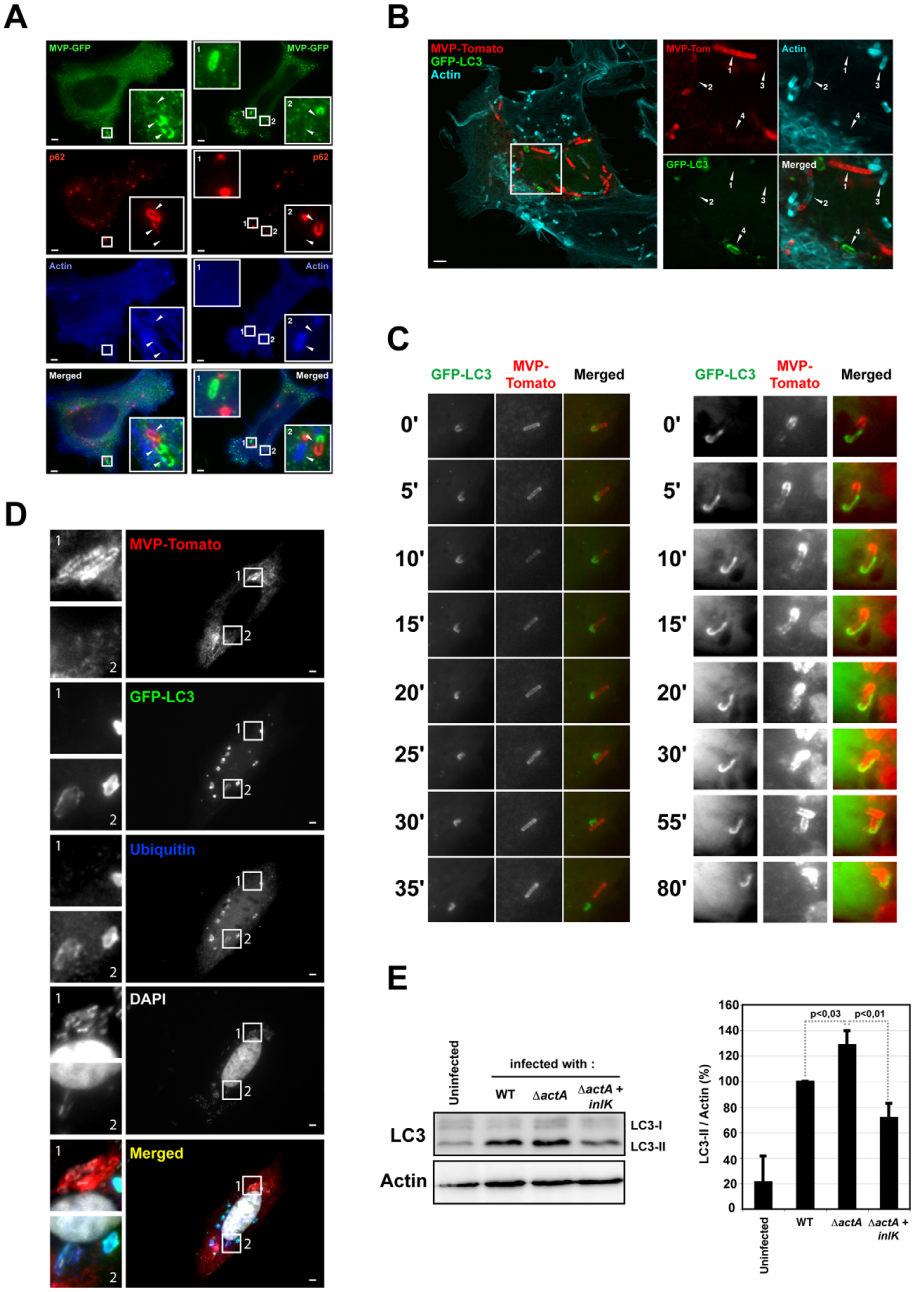
MVP impairs the recruitment of autophagy markers. A. Impaired recruitment of p62 to MVP positive *Listeria*. HeLa cells were transfected with MVP-GFP (green), infected with InlK over-expressing *Listeria* (Δ*inlK*+pPRT-*inlK*) for 4 h, fixed for fluorescence light microscopy, and stained with phalloidin (blue) and anti-p62 antibody (red). Inset regions are magnified. Arrows indicate independent bacteria The scale bar represents 1 µm. The vast majority of MVP-positive bacteria were completely devoid of anti-p62 labeling (95.1±2.0%; mean ± SEM from n = 3 experiments) but 4.9±2.0% (mean ± SEM from n = 3 experiments) were stained at one pole with MVP and at the other pole with p62. B. Impaired recruitment of GFP-LC3 on MVP positive *Listeria*. HeLa cells were transfected with MVP-tomato (red) and GFP-LC3 (green), infected with InlK over-expressing *Listeria* (Δ*inlK*+pPRT-*inlK*) for 4 h, fixed for fluorescence light microscopy, and stained with phalloidin (blue). Inset regions are magnified. The scale bar represents 1 µm. MVP and/or actin positive bacteria were never recognized by GFP-LC3. Arrows point to bacteria at different steps of the infection process: 1) InlK over-expressing bacterium is totally covered by MVP; 2) bacterium is partially labeled with MVP (at the poles) and actin (at the center); 3) bacterium is completely covered by actin; 4) bacterium is enclosed in an GFP-LC3 positive autophagosome. C. Kinetics of autophagy escape for MVP positive *Listeria*. Jeg3 cells were transfected with MVP-tomato (red) and GFP-LC3 (green), infected with InlK over-expressing *Listeria* (Δ*inlK*+pPRT-*inlK*) for 4 h, and prepared for real-time video microscopy. Image series were collected every 5 min for 2 h. Time is indicated along the Y axis. The left panel shows that the GFP-LC3 membranous aggregate detaches from the MVP positive *Listeria*. The entire image sequence can be viewed as Video S2. The right panel shows that the GFP-LC3 membranous aggregate on MVP positive bacteria does not lead to an autophagosome formation, whereas those bacteria efficiently divided. The entire image sequence can be viewed as Video S3. D. Impaired recruitment of GFP-LC3 and ubiquitin to MVP positive Δ*actA Listeria*. HeLa cells were transfected with MVP-tomato (red) and GFP-LC3 (green), infected with InlK over-expressing Δ*actA* (Δ*actA*+pADc-*inlK*) for 4 h, fixed for fluorescence light microscopy, and stained with anti-ubiquitin antibody (blue) and DAPI (white). Inset regions are magnified. The scale bar represents 1 µm. E. LC3 levels in infected RAW 267.4 macrophages. Left panel: RAW 267.4 macrophages were infected with *L. monocytogenes* EGD (WT), Δ*actA* or Δ*actA*+InlK for 6 h. Cell total lysates were immunobloted for LC3 and actin. Western blot is representative from 3 independent experiments. Right panel: Quantification of the relative LC3-II level (mean ± SEM) shown in the left panel. Statistical analyses were performed on the results of 3 independent experiments using the Student's *t* test. *P* values of <0.05 were considered statistically different.

To test if MVP recruitment could lead to autophagic escape, we used two well-established markers of autophagy, p62 (SQSTM1) and LC3 (Atg8) [54]. p62 has emerged as the prototypic adaptor involved in directing cytoplasmic substrates towards autophagic degradation [55]. p62 interacts with ubiquitinated subtrates *via* its ubiquitin-binding domain, and links them to the autophagosomal structural protein LC3. We infected MVP-transfected HeLa cells with InlK over-expressing bacteria for 4 h and after fixation, immuno-stained for endogenous p62 and actin. No co-localization could be observed between MVP and p62 (Figure 5A, Figure S4A) or MVP and LC3 (Figure 5B, Figure S4B). Interestingly, the vast majority of MVP-positive bacteria were completely devoid of anti-p62 labeling (95.1±2.0%; mean ± SEM from n = 3 experiments) but 4.9±2.0% (mean ± SEM from n = 3 experiments) were stained at one pole with MVP and at the other pole with p62. Similar results were obtained using GFP-LC3 (Figure 5, Figure S4B). As previously demonstrated [11], bacteria that had started to recruit actin were not labeled by p62 (Figure 5A) or GFP-LC3 (Figure 5B). Strikingly, when the MVP-positive bacteria that exhibited a recruitment of LC3 at one pole were examined by live-cell-imaging (Figure 5C), the membrane elongation leading to the autophagosome formation failed to occur (Video S2 and S3). Together these results indicate that bacteria which either recruit MVP or have started to polymerize actin evade autophagic recognition.

We thus studied autophagy marker recruitment by the *actA* mutant over-expressing InlK. In agreement with our previous observations (Figure S3D), InlK over-expressing Δ*actA* bacteria efficiently recruited MVP [88.6±12.8% (mean ± SEM from n = 3 experiments)] (Figure 5D and S4C). These MVP positive bacteria were neither surrounded by ubiquitinated proteins nor recognized by LC3 (Figure 5D and S4C). Furthermore, the level of LC3-II, the active form of LC3 that correlates with active autophagy [54], was significantly lower in cells infected with Δ*actA*+InlK as compared with Δ*actA* (1.82±0.14 fold) (Figure 5E). Together, these results show that in the absence of ActA, *Listeria* is able to evade autophagic recognition *via* MVP recruitment.

### MVP-dependent escape from autophagy leads to increased *Listeria* survival

**Figure 6:**
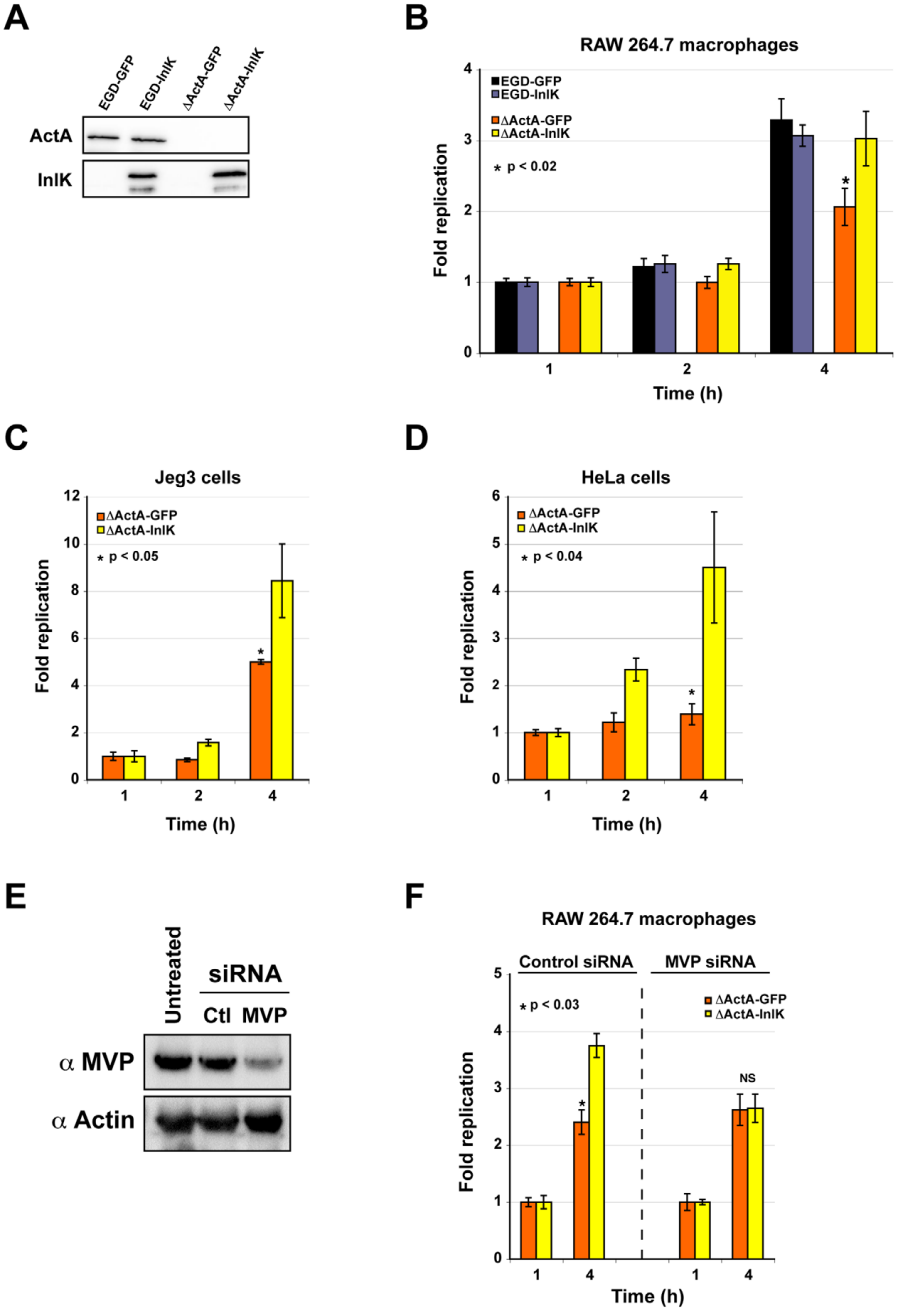
MVP-dependent escape from autophagy leads to increased *Listeria* survival. A. InlK and ActA expression in *Listeria* strains used for survival assays. Total lysates of *L. monocytogenes* EGD-(pADc-GFP), EGD-(pADc-*inlK*), Δ*actA*-(pADc-GFP) and Δ*actA*-(pADc-*inlK*) grown in BHI were immunoblotted using anti-ActA and anti-InlK antibodies. B. Intracellular survival of EGD-(pADc-GFP), EGD-(pADc-*inlK*), Δ*actA*-(pADc-GFP) and Δ*actA*-(pADc-*inlK*) in RAW 267.4 macrophages. Statistical analyses were performed on the results of 3 independent experiments using the Student *t* test. *P* values of <0.05 were considered statistically different and are labeled here as *. C. Intracellular survival of Δ*actA*-(pADc-GFP) and Δ*actA*-(pADc-*inlK*) in MVP-GFP transfected Jeg3 cells. Statistical analyses were performed on the results of 3 independent experiments using the Student's *t* test. *P* values of <0.05 were considered statistically different and are labeled here as *. D. Intracellular survival of Δ*actA*-(pADc-GFP) and Δ*actA*-(pADc-*inlK*) in MVP-GFP transfected HeLa cells. Statistical analyses were performed on the results of 3 independent experiments using the Student's *t* test. *P* values of <0.05 were considered statistically different and are labeled here as *. E. MVP levels in RAW 267.4 macrophages treated with MVP-siRNA. Western blot is representative from 3 independent experiments. F. Intracellular survival of Δ*actA*-(pADc-GFP) and Δ*actA*-(pADc-*inlK*) in MVP knock-down RAW 267.4 macrophages. Statistical analyses were performed on the results of 3 independent experiments using the Student *t* test. *P* values of <0.05 were considered statistically different and are labeled here as *.

Autophagy is recognized as a cell-autonomous innate defense mechanism that may control growth of intracellular microbes [56]. We thus tested if MVP-mediated autophagy escape leads to increased bacterial survival. As macrophages are among the cells which express the highest levels of MVP [29], [57], [58], the intracellular survival of WT, WT+InlK, Δ*actA* and Δ*actA*+InlK was analysed in RAW 264.7 macrophages (Figure 6A). These four strains (WT, WT+InlK, Δ*actA* and Δ*actA*+InlK) were first verified to grow identically in culture medium (data not shown). As previously described by Yoshikawa et al. [11], the intracellular survival rate of Δ*actA* bacteria at 4 h post-infection was significantly lower than that of WT bacteria (Figure 6B). Strikingly, the expression of InlK by the Δ*actA* strain restored the intracellular survival rate to the level of WT bacteria (Figure 6B), indicating that InlK could functionally replace ActA in its role in autophagy escape. Infection of MVP-transfected epithelial cells with Δ*actA* and Δ*actA*+inlK led to similar results (Figure 6C and 6D).

The intracellular survival of Δ*actA* and Δ*actA*+InlK was then analysed in RAW 264.7 macrophages treated with control or MVP siRNA (Figure 6E). As previously observed (Figure 6B), the Δ*actA*+InlK strain replicated better than the Δ*actA* strain in control cells (Figure 6E). Strikingly, in MVP-depleted cells, the Δ*actA*+InlK strain did not replicate faster than the Δ*actA* strain (Figure 6F), confirming the role of InlK/MVP interaction in survival rate. Taken together, these data show that the specific recruitment of MVP to the bacterial surface *via* InlK leads to a better survival of *L. monocytogenes*.

## Discussion

*L. monocytogenes* has emerged as a paradigm to study host-pathogen interactions and fundamental processes in cell biology [5], [59]. However the role of the many proteins expressed on the bacterial surface during *Listeria* infection remains fragmentary [15]. In this study we report that InlK, a *L. monocytogenes* surface protein of the internalin family, plays a critical role in *Listeria* virulence. We show that InlK is anchored to the listerial surface through its LPXTG peptidoglycan anchoring signal by sortase A and is produced during *in vivo* infection, whereas it cannot be detected on bacteria grown in BHI medium [44] or within the cytosol of tissue-cultured cells. This *in vivo* specific expression profile was previously described for other virulence factors of *L. monocytogenes*, e.g. the internalin InlJ, that behaves as an adhesin [49] and recently LntA, a secreted bacterial protein involved in chromatin remodeling and type III interferon response [60]. Furthermore, our results confirm and extend our recently published transcriptomic analysis of *L. monocytogenes* [43] which identified *inlK* as a gene highly activated during *in vivo* infection and that may play a role in the infectious process. Together, our results demonstrate that InlK is a so far undescribed virulence factor of *L. monocytogenes*.

To enter, survive and spread from cell-to-cell, *L. monocytogenes* has been shown to interact with several host partners. We revealed here that MVP is a specific cellular interactor of InlK. The highly conserved MVP protein constitutes more than 70% of the mass of the largest cytoplasmic ribonucleoprotein (RNP) complex known, i.e. vault particles [16], [18], [19]. Since its first description in 1986 [61], several putative functions have been attributed to this RNP complex. Data that link the vault complex to various functions have suggested roles in multidrug resistance [28], [62], transport [63], signaling [28], [32]–[35], apoptosis resistance [33], [36] or innate immunity [30]. However, no compelling evidence for a cellular role was reported unequivocally and MVP was mainly considered as a scaffold protein. Nevertheless, vaults were previously found to be implicated in g-herpesvirus (Epstein-Barr and Kaposi's sarcoma virus) [31], [64] and *Pseudomonas aeruginosa* infectious processes [30]. During Epstein-Barr or Kaposi's sarcoma virus infection, the expression of vault RNAs (vRNAs) was shown to be specifically up-regulated in human lymphocytes [31], [64]. However, the function of this overexpression was not assessed. In addition, not only vRNA but also MVP was reported to be upregulated during viral infection by human T-cell lymphotropic virus type I (HTLV-I) infection [65]. In the case of bacteria, MVP was implicated in host resistance to *P. aeruginosa* lung infection [30]. Indeed, a rapid recruitment of MVP to lipid rafts was observed when human lung epithelial cells were infected with *P. aeruginosa*. This recruitment was dependent on bacterial binding to the cystic fibrosis transmembrane conductance regulator CFTR. However, no evidence of direct binding between MVP and bacteria was observed. Our results provide the first report of a direct interaction between a microbial protein and a component of the vault particles. Indeed, we demonstrated that InlK over-expressing *L. monocytogenes* were able to directly bind MVP. In agreement with previous observations that MVP/vaults are predominantly (>90%) localized in the cytoplasm [28], [51], [52], we established that the InlK/MVP interaction occurs in the cytosol of infected cells, after the disruption of the internalization vacuole, and independently of actin polymerization.

**Figure 7:**
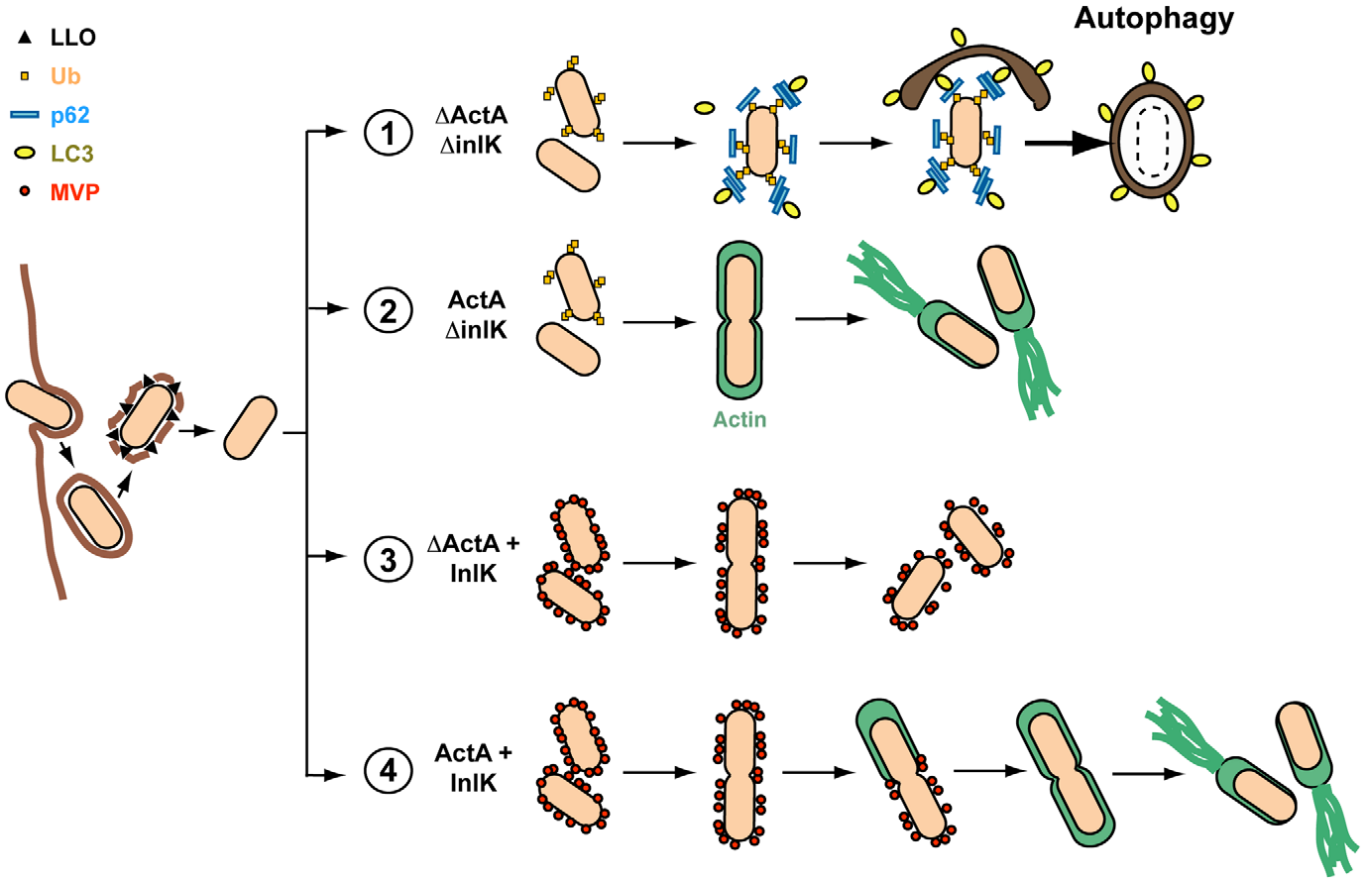
Model for escape of autophagic recognition for *L. monocytogenes* expressing InlK. During intracellular growth, cytoplasmic bacteria are able to escape from autophagy process using two independent virulence factors, ActA and InlK. On the one hand, the recruitment of VASP, Arp2/3 complex and actin *via* ActA masks the bacteria from ubiquitination and autophagic recognition. On the other hand, MVP recruitment *via* InlK is also able to protect bacteria from ubiquitination and autophagic recognition. In that way, depending on ActA and InlK expression, four possibilities could be distinguished: (1) When neither ActA nor InlK are expressed, the bacterial ubiquitination is followed by p62 and LC3 recruitment, leading to autophagosome formation around the bacterium. (2) When ActA is expressed (e.g. wild-type bacterium (WT) grown in BHI before cell infection) it is sufficient for *Listeria* to escape from autophagy. (3) In contrast, in the absence of ActA, InlK efficiently protects bacterium against autophagy recognition *via* MVP recruitment. (4) Finally, when ActA and InlK are co-expressed by the bacterium, InlK rapidly recruits MVP at the surface of the bacterium. Then, in some instance, ActA replaces InlK leading to a switch of the bacteria disguised from MVP to actin. The model is partially based on the results of Yoshikawa *et al* [11].

As with a variety of intracellular microbes, intracytosolic *L. monocytogenes* are recognized by autophagy, a cell-autonomous effector mechanism of innate immunity that protects the cytosol against bacterial invasion [66]. Perrin *et al*. first demonstrated that cytosolic *L. monocytogenes* occasionally colocalized with ubiquitin in infected cells, and this association was more frequent in case of the Δ*actA* strain [13], [14]. More recently, Yoshikawa *et al*. demonstrated that the recruitment of VASP, Arp2/3 complex and actin *via* ActA protect bacteria from ubiquitination and autophagic recognition [11]. Here we reveal that *L. monocytogenes* has a second strategy to escape autophagy in the absence of ActA (Figure 7). Indeed, no significant difference could be observed between the intracellular survival rate of WT and WT+InlK bacteria in infected RAW 267.4 macrophages (Figure 6B), suggesting that when ActA is expressed it is sufficient for *Listeria* to escape from autophagy. In contrast, in absence of ActA, InlK protects against autophagy. Together, our results show that the bacteria are able, *via* InlK, to decorate their surface with MVP in order to escape from autophagy (Figure 7). It will be thus of the highest importance to decipher in which cells InlK is expressed *in vivo* and when the InlK/MVP interaction takes place during infection. These data will be critical to unravel the role of InlK in the pathophysiology of *Listeria* infection. It will also be of great interest to further study the link between actin polymerization, MVP, autophagy, and pathogen dissemination.

## Materials and Methods

### Bacterial strains, growth conditions and reagents

*Listeria* strains (Table S2) were grown in brain-heart infusion (BHI) medium (Difco; BD) and *Escherichia coli* were grown in Luria-Bertani Medium (LB) medium (Difco; BD). When required, chloramphenicol and erythromycin were used at final concentrations of 7 µg/ml and 5 µg/ml respectively for *L. monocytogenes* and kanamycin, erythromycin and chloramphenicol were used at final concentration of 50 µg/ml, 150 µg/ml and 35 µg/ml, respectively for *E. coli*.

### Generation of EGD-e Δ*inlK* mutant strain and inlK over-expressing strains

#### Generation of Δ*inlK* mutant strain

Two ∼700 pb fragments flanking *inlK* gene were PCR amplified from EGD-e chromosomal DNA. The primers used for the *inlK* 5′ flanking fragment were A (5′-TTG GAT CCG CTG TAG ATT TCA CAA AAG-3′) and B (5′-TAA CAC GCG TAA GTC ATT ATC CTC TCC ACT C-3′), and the primers used for the 3′ fragment were C (5′-GAA AAC GCG TAA AAA ACT ATC CGC CCA C-3′) and D (5′-TTG GTC CAT GGT TAA GCA TTG CTG GTG-3′). After restriction of the amplified 5′ and 3′ fragments with *Bam*HI and *Mlu*I, and *Mlu*I and *Nco*I respectively, 5′ and 3′ fragments were coligated in the thermosensitive plasmid pMAD [67] digested by *Bam*HI and *Nco*I, yielding the pMAD-Δ*inlK* plasmid. The sequence was verified by sequencing. This plasmid was electroporated into *L. monocytogenes* EGD-e. Independent colonies were used for allelic exchange in *L. monocytogenes* wild-type EGD-e, which was performed as previously described [49], generating a Δ*inlK* isogenic deletion mutant (Table S2). Deletion of the entire *inlK* gene was confirmed by PCR amplification and sequencing.

#### Generation of InlK over-expressing strains

To express InlK in *L. monocytogenes* the pPRT- and pADc- derivative plasmids were constructed as described below. In the pPRT*-inlK* plasmid, *inlK* was expressed under the control of the promoter region of the protease gene from *Lactococcus lactis* subsp. *cremoris*, which is active in *Listeria* [49]. This is a multicopy plasmid which expresses an erythromycin resistance gene used for cloning selection.

The pADc-*inlK* plasmid generated as previously described by Balestrino et al [48] was derived from the integrative pPL2 plasmid, which inserts in the *Listeria* chromosome at the tRNAArg-*attBB* site, thereby avoiding the requirement for antibiotic pressure to maintain the plasmid and preventing heterogeneity of InlK expression due to variation in the plasmid copy number.

### Cell lines and infection

HeLa cells (human epithelial cervix carcinoma; ATCC CCL2), Jeg3 cells (human placenta choriocarcinoma, ATCC HTB-36) and RAW 267.4 murine macrophages (ATCC TIB-71) were grown as recommended by ATCC (Manassas, VA). Cells were infected with exponentially growing *Listeria* strains such that the multiplicity of infection was 50 bacteria per cell (MOI_50_) for epithelial cell lines and MOI_10_ for RAW 267.4 macrophages. After 1 h of infection for epithelial cell lines and 15 min for RAW 267.4 macrophages, cells were washed and treated with 25 µg/ml of gentamicin. Incubation times were as indicated. All experiments were performed in serum-free medium. Then, cells were washed three times with PBS 1X (Difco, BD) and lysed by adding 500 µl of 0.1% Triton X-100. The number of viable bacteria released from cells was assessed by plating serial dilutions of bacteria on agar plates.

### siRNA experiments

2.5×10^5^ RAW 267.4 macrophages per well were plated in 12 wells plates and incubated at 37°C in 10% CO_2_. 24 h after plating, cells were treated with 80 nM of either a pool of anti-mouse MVP siRNA (ON-TARGETplus SMART pool L-049201-01-005 Mouse MVP, Dharmacon) or control siRNA (ON-TARGETplus Non-targeting siRNA:1, Dharmacon), using Lipofectamine 2000 (Invitrogen) as recommended by the manufacturer. The following day, the medium was changed and the cells were incubated in complete medium for another 24 hours. Infections were performed as above-mentioned and the efficiency of siRNA knock-down was assessed by performing Western-blot on total cell lysates in each experiment (Figure 6E).

### InlK purification

The *inlK* coding sequence (aa 27–568) was amplified using primers *lmo1290*-Fw: 5′- GAG TCG GAT CCG GTA TTT GCT GCA GAG CAAC C-3′ and *lmo1290*-Rev: 5′- GAG TCG TCG ACA GCC TCT TTA CTT GGT TCT G-3′. The PCR product was purified and ligated with pET28b (Novagen) plasmid after double digestion with *Bam*H*I* and *SalI* enzymes. The ligation product was electroporated in *E.coli* XL-1 Blue and positive clones were selected on 50 µg/ml supplemented BHI and sequenced (BUG 2812). For purification, *E.coli* BL21(DE3) (Invitrogen) were chemically transformed with the purified His_6_-InlK-His_6_ expressing pET28b plasmid. Bacteria were grown in 50 µg/ml supplemented LB until OD_600_ 0.6 and IPTG was added at the final concentration 1 mM for 4 additional hours. Bacteria were lysed using a French press and the supernatant was recovered. His_6_-InlK-His_6_ purification was performed using TALON His-Tag Purification Resins (Clontech). Increased concentration of imidazole (0–200 mM) in Tris-HCl 20 mM, NaCl 0.5 M (pH = 8) were used for purification and elution of InlK. The purified protein was dialysed against Tris-HCl 20 mM, NaCl 0.5 M (pH = 8) buffer and concentrated using AmiconUltra centrifugal filter (Millipore).

### Antibodies and reagents

The primary antibodies used in this study were anti-actin mouse monoclonal (mAb) (AC-15; Sigma-Aldrich), anti-LRP mAb (MVP was also named LRP for Lung resistance protein) (Ref:610512; BD Biosciences), anti-p62 mAb (Ref:610832, BD Biosciences), anti-ubiquitin mAb (FK-2, Affiniti), anti-Atg8 (LC3) rabbit polyclonal (pAb) (Novus Biologicals, Ref:NB100-2331), anti-killed *L. monocytogenes* pAb (R11), anti-live *L. monocytogenes* pAb (R177). Monoclonal antipeptide antibody that recognizes ActA (A16) was obtained as previously described [68]. An anti-InlK polyclonal rabbit antibody (R190) was generated against His_6_-InlK-His_6_ recombinant protein (aa 27–568) deleted from its signal peptide and peptidoglycan-anchoring sequence and affinity-purified on a ECH Sepharose 4B column (GE Healthcare) coupled with 2.5 mg His_6_-InlK-His_6_ recombinant protein expressed from pET28b-InlK plasmid as described above. The polyclonal pre-immune serum of R190 (pre-immune R190) was recovered from rabbits before they were s.c injected with purified InlK. The secondary antibodies were Alexa Fluor 488- and 546-conjugated goat anti-mouse and anti-rabbit, respectively (Molecular Probes) and HRP-conjugated goat anti-mouse and goat anti-rabbit (AbCys). Alexa fluor 647-conjugated phalloidin was purchased from Molecular Probes; DAPI from Roche Applied Sciences; and the Amersham ECL Plus kit from Ge Healthcare.

The GST-tagged purified recombinant MVP protein was purchased from Abnova (Ref:H00009961-P01).

### Immunofluorescence microscopy

Cells were fixed in 4% paraformaldehyde (PFA) in 1X PBS for 20 minutes at room temperature. Cells were then rinsed in 1X PBS before incubation in blocking solution (0.5% BSA, 50 mM NH4Cl in PBS, pH 7.4) containing 0.05% saponin. Cells were then incubated with the primary antibodies diluted in the blocking solution for 30 min at room temperature, rinsed 5 times in 1X PBS and further incubated for 30 minutes with the secondary antibodies diluted in blocking solution. Where needed, fluorescent phalloidin was added with the secondary antibodies to label actin. Cells were then rinsed 5 times in 1X PBS and mounted on glass coverslip using Fluoromount mounting medium (EMS, PA). The differential immuno-staining between extra- and intracellular *Listeria* was previously described [69]. Samples were analysed either with a Zeiss Axiovert 135 epifluorescence microscope connected to a CCD camera or with a Zeiss LSM510 confocal microscope (Carl Zeiss, Germany). Images were acquired with a 100X oil immersion objective and images were processed using MetaMorph (Universal Imaging Corp.).

### Plasmids

The MVP-GFP plasmid that encodes EGFP fused to MVP C-terminus has been previously described [70]. To construct MVP-CFP (BUG 2908) and MVP-Tomato (BUG 2909), the MVP coding sequence was isolated from the MVP-GFP (BUG 2907) plasmid by double enzyme digestion (*Hin*dIII and *Bam*HI) and ligated in pECFP-N1 and ptdTomato-N1. Briefly, ptdTomato-N1 was constructed by replacing EGFP, from pEGFP-N1 vector (Invitrogen), by tdTomato, from ptdTomato-LCa vector (BUG 2420) [69]. Plasmid encoding CBD-YFP (BUG 2305) [53], actin-GFP (BUG 2421), actin-CFP (BUG 2155) and GFP-LC3 (BUG 3046) [71] were described elsewhere. Cells transfections were performed with FuGENE HD (Roche) as recommended by the manufacturer.

### Bacterial pull down assay

To test the binding of bacteria to GST-MVP, *L. monocytogenes* strains were grown in BHI to an OD_600_ of 1.0, and 1 ml of each culture was taken for each reaction. Bacterial cells were washed twice in buffer with 20 mM HEPES pH 7.5, 150 mM NaCl, resuspended in 1 ml of binding buffer (20 mM HEPES pH 7.5, 150 mM NaCl, 1 mM CaCl2, 1 mM MgCl2, and 0.2% BSA), and incubated at room temperature on a rotating wheel for 30 min. GST-MVP recombinant protein was added to a final concentration of 1 µg/ml and samples were incubated with rotation for an additional hour. Samples were centrifuged and pellets were washed three times in 20 mM HEPES pH 7.5, 300 mM NaCl, 0.05% Tween 20 and three times in buffer lacking Tween 20. The final bacterial pellets were resuspended in 20 microliters of 2X sample buffer, boiled for 10 min, and stored at −20°C before migration on 8% SDS/PAGE gels and Western blotting.

To analyse the binding of bacteria to transfected MVP-GFP HeLa cell lysates, HeLa cells were grown on 75 cm^2^ flask, then transfected with MVP-GFP plasmid 24 h prior to the experiment. Cells were lysed at 4°C for 10 min in a 1 ml of lysis buffer (Tris-HCl 50 mM, pH 7.5, NaCl 150 mM, EDTA 2 mM, NP40 1%, AEBSF 1 mM, Na_3_VO_4_ 3 mM). Cells were scraped and the lysates were incubated with rotation for additional 15 min. Lysates were cleared by 15 min centrifugation at 13 000 g at 4°C. *L. monocytogenes* strains were grown in BHI to an OD_600_ of 1.0, and 1 ml of each culture was taken for each reaction. Bacterial cells were washed twice in lysis buffer and were resuspended in cell lysate supernatants, mainly corresponding to cytoplasmic fraction, and incubated at room temperature on a rotating wheel for 1 hour. Samples were centrifuged to pellet bacteria, and washed five times in washing buffer (Tris-HCl 50 mM, pH 7.5, NaCl 150 mM, EDTA 2 mM, NP40 0.2%, AEBSF 1 mM, Na_3_VO_4_ 3 mM). The final bacterial pellets were treated as described above.

In each experiments bacterial inoculi were counted to ensure that an equal number of bacteria were used for pull-down assay.

### Co-immunoprecipitation

HT29 (colon epithelial cells, ATCC HTB-38) cells were cultured to confluence in 75 cm^3^ flask. Cells were lysed for 10 min at 4°C in 1 ml of lysis buffer (Tris-HCl 50 mM, pH 7.5, NaCl 150 mM, EDTA 2 mM, NP40 1%, AEBSF 1 mM, Na_3_VO_4_ 3 mM). Cells were scraped and the lysates were incubated with rotation for additional 15 min. Lysates were cleared by 15 min centrifugation at 13 000 g at 4°C and were incubated overnight at 4°C with 20 µg of purified InlK. Then, co-immunoprecipitation was performed using 1 µg of anti-MVP antibody. After five washes in lysis buffer, samples were resuspended in 20 microliters of 2X sample buffer, boiled for 10 min, and stored at −20°C before migration on 8% SDS/PAGE gels and Western blotting.

Stable HEK293-HTP Blue and HEK293-HTP InlK were constructed as previously described [60]. When necessary cells were treated with doxycyclin 24 h prior the assay. Co-immunoprecipitations were performed using anti-MVP antibody as described above.

### Yeast two-hybrid screening

The InlK encoding sequence (aa 27–568) was amplified by PCR from EGD-e and cloned into pB29 (N-bait-LexA-C fusion) plasmid. Randomly primed cDNA from human placenta poly(A) were constructed into a prey plasmid derived from pBTM116. The two-hybrid screen was performed by Hybrigenics (www.hybrigenics.com). The DNA inserts of the positive clones were sequenced to identify the corresponding gene in GenBank database using a fully automated procedure. Results of the yeast two-hybrid screening are recapitulated in Table S1.

### Immunoblotting

Cells were seeded into 6 well plates and incubated 24 h before infection. Infections were done as described above. At the indicated times cells were lysed and lysates were analysed by Western blot. The immunoblots shown are representative of three independent experiments. Analysis of immunoblots was performed using G:Box Gel documentation system (Syngene).

### Murine infection experiments

All experiments were performed according to Institut Pasteur guidelines for laboratory animal husbandry. For determination of LD50, groups of 8-week-old BALB/c female mice (Charles River Laboratory) were injected i.v with increasing concentrations of *L. monocytogenes* WT strain or D*inlK* mutant. LD50 were determined by the probit method at 10 days after inoculation.

Bacterial growth in mice was determined by injecting 6- to 8-week-old female BALB/c mice intravenously with a sublethal bacterial inoculum, 10^4^ CFU. After 24, 48 72 and 96 h of infection, liver and spleen were dissected in sterile conditions and the numbers of CFU were determined by plating serial dilutions of organ (liver and spleen) homogenates on BHI agar medium.

### Ethics statement

All animals were handled in strict accordance with good animal practice as defined by the relevant national and local animal welfare bodies, and all animal work was approved by the Institut Pasteur animal experimentation committee which comply with European regulations (directive 2010/63/EU of the European parliament and of the council of 22 September 2010 on the protection of animals used for scientific purposes).

## Supporting Information

Figure S1 ***In vitro*** **,** ***in cellulo*** **and** ***in vivo*** **expression of** ***inlK.*** A. Coomassie staining of purified InlK recombinant protein and bovine serum albumine (BSA). B. *In cellulo* and *in vitro* expression of *inlK* revealed by bioluminescence. Left panel: RAW 267.4 macrophages and HeLa epithelial cells were infected for 4 h with wild-type *L. monocytogenes* EGD-e that contain a bioluminescent reporter of either *inlK* promoter [EGD-e-(pPL2-P*_inlK_*-*lux_ABCDE_*)] or *hly* promoter [EGD-e-(pPL2-P*_inlK_*-*lux_ABCDE_*)], and submitted to photon detection with IVIS 100 (Xenogen/Caliper) system. Right panel: EGD-e-(pPL2-P*_inlK_*-*lux_ABCDE_*) and EGD-e-(pPL2-P*_inlK_*-*lux_ABCDE_*) were grown in BHI to OD_600_ 1.0 and submitted to photon detection with IVIS 100 (Xenogen/Caliper) system. C. *In vivo* expression of *inlK* revealed by bioluminescence. Left panel: Five BALB/c mice were i.v. infected with either EGD-e-(pPL2-P*_inlK_*-*lux_ABCDE_*) or EGD-e-(pPL2-P*_inlK_*-*lux_ABCDE_*). Each 24 h mice were anesthetized and submitted to photon detection with IVIS 100 (Xenogen/Caliper) system. Right panel:Quantification of the CFU number recovered from livers and spleens of infected mice, 72 h post infection. NS = No significant difference.
(TIF)

Figure S2 **InlK interacts with MVP.** A. Bacterial pull-down of MVP-GFP from transfected HeLa cell lysates with the *L. monocytogenes* strains EGD Δ*actA*-(pADc-GFP), EGD Δ*actA*-(pADc-*inlK*) and EGD-e Δ*inlK*-(pPRT-*inlK*). MVP-GFP bound to InlK over-expressing bacteria but not to other bacteria. B. Co-immunoprecipitation of InlK and endogenous MVP in stable HEK293 cells. Control HEK293 (HEK293-HTP-Blue) and InlK expressing HEK293 (HEK293-HTP-InlK) were treated by doxycycline to induce InlK expression 24 h prior of co-immunoprecipitation with anti-MVP antibody.
(TIF)

Figure S3 **InlK/MVP interaction occurs in the cytosol, independently of actin polymerization.** A. Scheme of CBD-YFP recruiting bacteria during *L. monocytogenes* intracellular cell cycle. The image is based on Henry et al results [44]. B. Detection of MVP recruitment at the surface of InlK over-expressing bacteria that do no recruit actin. HeLa cells were transfected with MVP-GFP (green) and actin-CFP (yellow), infected with InlK over-expressing *Listeria* (Δ*inlK*+pPRT *inlK*) for 4 h, fixed for fluorescence light microscopy, and stained with anti-InlK (blue) and anti-ActA (red) antibodies. MVP-GFP and actin-CFP and their respective bacterial interactors, InlK and ActA, are never co-recruited Inset regions are magnified. Inset region 1 represents an MVP-GFP positive bacterium which is also labeled for InlK but not for actin-CFP and ActA. Opposingly, the inset 2 represents a bacterium that recuits actin-CFP which is also labeled for ActA, but not for MVP-GFP and InlK. The scale bar represents 1 µm. C. Detection of MVP recruitment at the surface of intracytosolic InlK over-expressing bacteria. HeLa cells were transfected with MVP-CFP (red) and YFP-CBD (green), infected with InlK over-expressing *Listeria* (Δ*inlK*+pPRT *inlK*) for 4 h, fixed for fluorescence light microscopy and stained with phalloidin (blue). MVP positive bacteria were also labeled with YFP-CBD revealing that MVP was recruited by intracytosolic bacteria after the lysis of the internalization vacuole. Inset regions are magnified. The scale bar represents 1 µm. D. Detection of MVP recuitment at the surface of intracytosolic Δ*actA* and InlK over-expressing Δ*actA Listeria*. HeLa cells were co-transfected with MVP-tomato (red) and CBD-YFP (green), infected with Δ*actA* or Δ*actA*-(pADc-*inlK*) for 4 h, and fixed for fluorescence light microscopy. Inset regions are magnified. The scale bar represents 1 µm. The percentage of intracytosolic D*actA* over-expressing InlK having recruited MVP at 4 h post-infection was 88.3±12.7% versus no recuitment for Δ*actA*.
(TIF)

Figure S4 **MVP and autophagy markers do not co-localize.** A. Impaired recruitment of p62 to MVP positive *Listeria*. HeLa cells were transfected with MVP-GFP (green), infected with InlK over-expressing *Listeria* (Δ*inlK*+pPRT *inlK*) (left panel) or Δ*inlK Listeria* (right panel) for 4 h, fixed for fluorescence light microscopy, and stained with phalloidin (blue) and anti-p62 antibody (red). Inset regions are magnified. Arrows indicate independent bacteria The scale bar represents 1 µm. The vast majority of MVP-positive bacteria were completely devoid of anti-p62 labeling (95.1±2.0%; mean ± SEM from n = 3 experiments) but 4.9±2.0% (mean ± SEM from n = 3 experiments) were stained at one pole with MVP and at the other pole with p62. B. Polar recruitment of GFP-LC3 to MVP positive *Listeria*. HeLa cells were transfected with MVP-tomato (red) and GFP-LC3 (green), infected with InlK over-expressing *Listeria* (Δ*inlK*+pPRT *inlK*) for 4 h, fixed for fluorescence light microscopy. Four different bacteria are shown. The scale bar represents 1 µm. C. Quantification of MVP and LC3 recruitment at the surface of Δ*actA* overexpressing InlK (mean%±SEM%). Quantifications correspond to the data represented in Figure 5D. The percentages MVP+/LC3+ bacteria, MVP+/LC3-, MVP-/LC3+ and MVP-/LC3- were 88.6±12.8%, 0.0±0.0%, 8.2±9.3% and 3.1±3.5% respectively. Statistical analyses were performed on the results of 3 independent experiments using the Student's *t* test.
(TIF)

Table S1 ***L. monocytogenes*** **strains used in this study.**
(DOC)

Table S2 **Results of the yeast two-hybrid screening.** The *L. monocytogenes* wild-type [72] and the Δ*actA* [73] were previously published.
(DOC)

Video S1 **Kinetics of MVP and actin recruitment at the surface of InlK over-expressing bacteria.** HeLa cells were transfected with MVP-tomato (red) and actin-GFP (green), infected with InlK over-expressing *Listeria* (Δ*inlK*+pPRT-*inlK*) for 4 h, and prepared for real-time video microscopy. Image series were collected every 15 min for 2 h.
(AVI)

Video S2 **Kinetics of autophagy escape for MVP positive** ***Listeria*** **.** Jeg3 cells were transfected with MVP-tomato (red) and GFP-LC3 (green), infected with InlK over-expressing *Listeria* (Δ*inlK*+pPRT-*inlK*) for 4 h, and prepared for real-time video microscopy. Image series were collected every 5 min for 2 h. The video shows that the GFP-LC3 membranous aggregate detaches from the MVP positive *Listeria*.
(AVI)

Video S3 **Kinetics of autophagy escape for MVP positive** ***Listeria*** **.** Jeg3 cells were transfected with MVP-tomato (red) and GFP-LC3 (green), infected with InlK over-expressing *Listeria* (Δ*inlK*+pPRT-*inlK*) for 4 h, and prepared for real-time video microscopy. Image series were collected every 5 min for 2 h. The video shows that the GFP-LC3 membranous aggregate on MVP positive bacteria does not lead to an autophagosome formation, whereas those bacteria efficiently divided.
(AVI)
