# Correction: Recruitment of the Major Vault Protein by InlK: A *Listeria monocytogenes* Strategy to Avoid Autophagy

**DOI:** 10.1371/annotation/a70544fc-6d8b-4549-921a-9e86557b0ffc

**Published:** 2011-09-13

**Authors:** Laurent Dortet, Serge Mostowy, Ascel Samba-Louaka, Edith Gouin, Marie-Anne Nahori, Erik A.C. Wiemer, Olivier Dussurget, Pascale Cossart

The third author's name was spelled incorrectly. The correct name is: Ascel Samba-Louaka. The correct citation is: Dortet L, Mostowy S, Samba-Louaka A, Gouin E, Nahori M-A, et al. (2011) Recruitment of the Major Vault Protein by InlK: A Listeria monocytogenes Strategy to Avoid Autophagy. PLoS Pathog 7(8): e1002168. doi:10.1371/journal.ppat.1002168

