# Supplementary figures and images for: Recruitment of the Major Vault Protein by InlK: A Listeria monocytogenes Strategy to Avoid Autophagy
Source: PLoS Pathog. 2011 Aug 4;7(8):e1002168. doi: 10.1371/journal.ppat.1002168 (PMC3150275; doi:10.1371/journal.ppat.1002168)

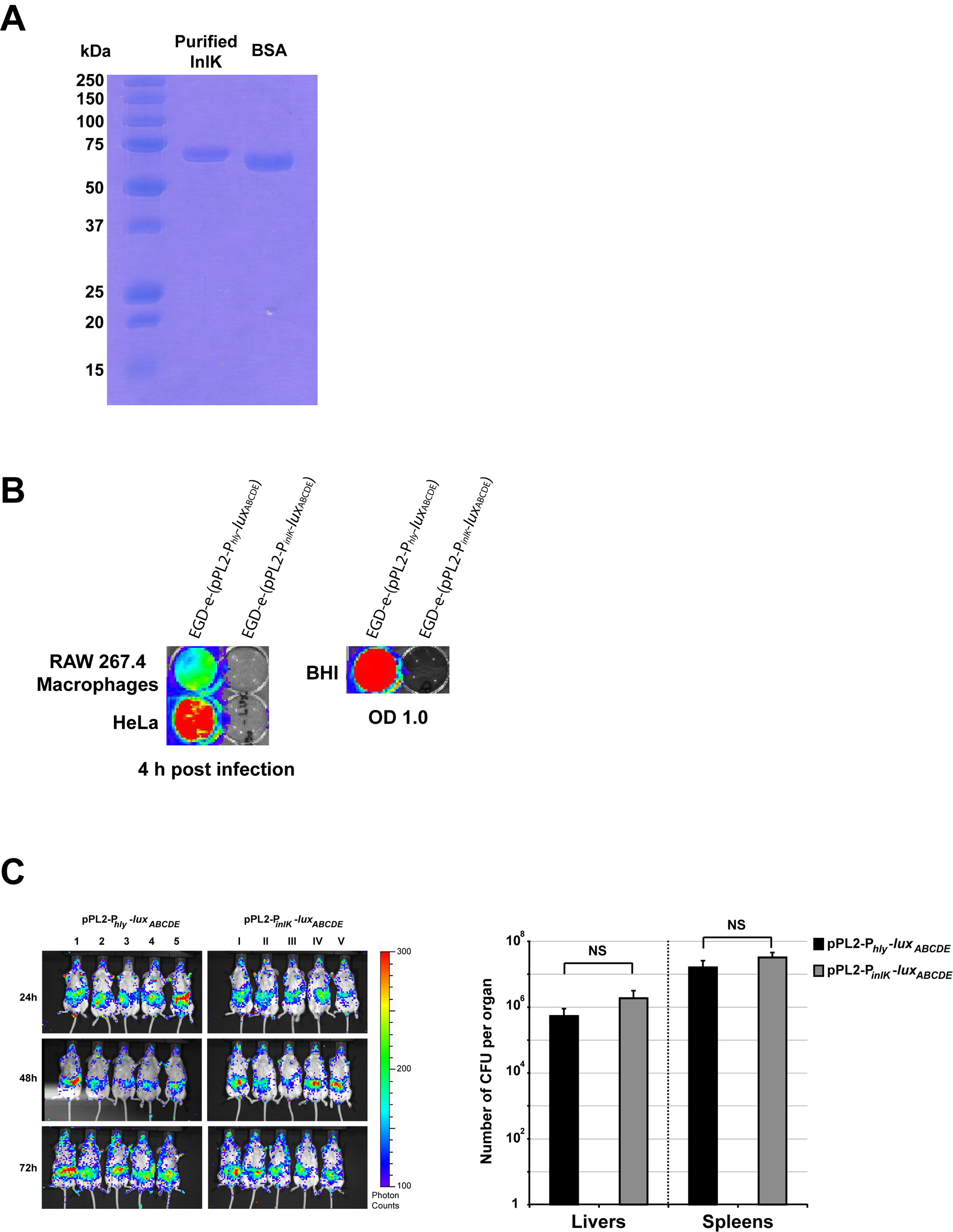

Supplement: Figure S1 — In vitro , in cellulo and in vivo expression of inlK. A. Coomassie staining of purified InlK recombinant protein and bovine serum albumine (BSA). B. In cellulo and in vitro expression of inlK revealed by bioluminescence. Left panel: RAW 267.4 macrophages and HeLa epithelial cells were infected for 4 h with wild-type L. monocytogenes EGD-e that contain a bioluminescent reporter of either inlK promoter [EGD-e-(pPL2-PinlK-luxABCDE)] or hly promoter [EGD-e-(pPL2-PinlK-luxABCDE)], and submitted to photon detection with IVIS 100 (Xenogen/Caliper) system. Right panel: EGD-e-(pPL2-PinlK-luxABCDE) and EGD-e-(pPL2-PinlK-luxABCDE) were grown in BHI to OD600 1.0 and submitted to photon detection with IVIS 100 (Xenogen/Caliper) system. C. In vivo expression of inlK revealed by bioluminescence. Left panel: Five BALB/c mice were i.v. infected with either EGD-e-(pPL2-PinlK-luxABCDE) or EGD-e-(pPL2-PinlK-luxABCDE). Each 24 h mice were anesthetized and submitted to photon detection with IVIS 100 (Xenogen/Caliper) system. Right panel:Quantification of the CFU number recovered from livers and spleens of infected mice, 72 h post infection. NS = No significant difference. (TIF) [file ppat.1002168.s001.tif]

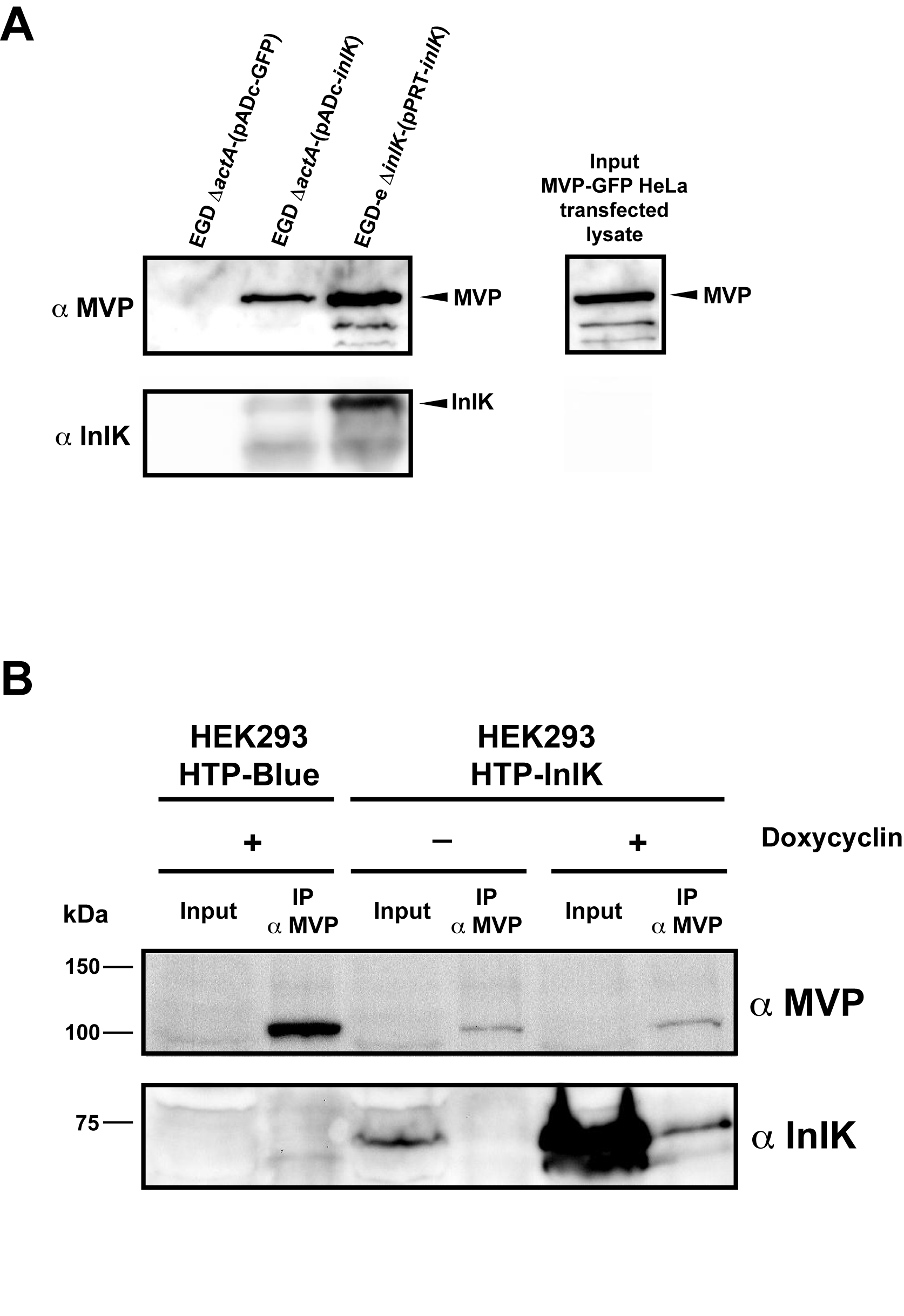

Supplement: Figure S2 — InlK interacts with MVP. A. Bacterial pull-down of MVP-GFP from transfected HeLa cell lysates with the L. monocytogenes strains EGD ΔactA-(pADc-GFP), EGD ΔactA-(pADc-inlK) and EGD-e ΔinlK-(pPRT-inlK). MVP-GFP bound to InlK over-expressing bacteria but not to other bacteria. B. Co-immunoprecipitation of InlK and endogenous MVP in stable HEK293 cells. Control HEK293 (HEK293-HTP-Blue) and InlK expressing HEK293 (HEK293-HTP-InlK) were treated by doxycycline to induce InlK expression 24 h prior of co-immunoprecipitation with anti-MVP antibody. (TIF) [file ppat.1002168.s002.tif]

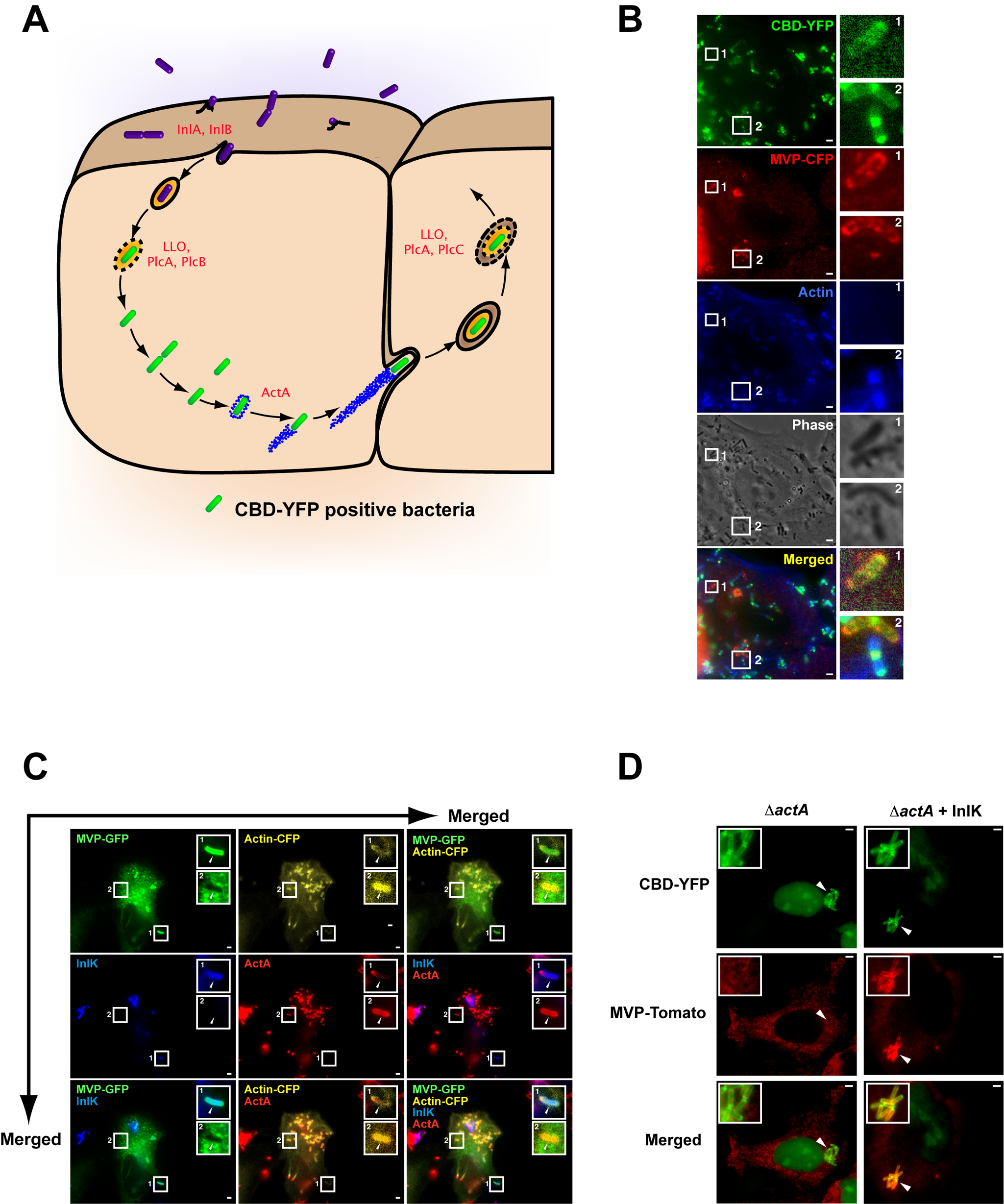

Supplement: Figure S3 — InlK/MVP interaction occurs in the cytosol, independently of actin polymerization. A. Scheme of CBD-YFP recruiting bacteria during L. monocytogenes intracellular cell cycle. The image is based on Henry et al results [44]. B. Detection of MVP recruitment at the surface of InlK over-expressing bacteria that do no recruit actin. HeLa cells were transfected with MVP-GFP (green) and actin-CFP (yellow), infected with InlK over-expressing Listeria (ΔinlK+pPRT inlK) for 4 h, fixed for fluorescence light microscopy, and stained with anti-InlK (blue) and anti-ActA (red) antibodies. MVP-GFP and actin-CFP and their respective bacterial interactors, InlK and ActA, are never co-recruited Inset regions are magnified. Inset region 1 represents an MVP-GFP positive bacterium which is also labeled for InlK but not for actin-CFP and ActA. Opposingly, the inset 2 represents a bacterium that recuits actin-CFP which is also labeled for ActA, but not for MVP-GFP and InlK. The scale bar represents 1 µm. C. Detection of MVP recruitment at the surface of intracytosolic InlK over-expressing bacteria. HeLa cells were transfected with MVP-CFP (red) and YFP-CBD (green), infected with InlK over-expressing Listeria (ΔinlK+pPRT inlK) for 4 h, fixed for fluorescence light microscopy and stained with phalloidin (blue). MVP positive bacteria were also labeled with YFP-CBD revealing that MVP was recruited by intracytosolic bacteria after the lysis of the internalization vacuole. Inset regions are magnified. The scale bar represents 1 µm. D. Detection of MVP recuitment at the surface of intracytosolic ΔactA and InlK over-expressing ΔactA Listeria. HeLa cells were co-transfected with MVP-tomato (red) and CBD-YFP (green), infected with ΔactA or ΔactA-(pADc-inlK) for 4 h, and fixed for fluorescence light microscopy. Inset regions are magnified. The scale bar represents 1 µm. The percentage of intracytosolic DactA over-expressing InlK having recruited MVP at 4 h post-infection was 88.3±12.7% versus no recuit [file ppat.1002168.s003.tif]

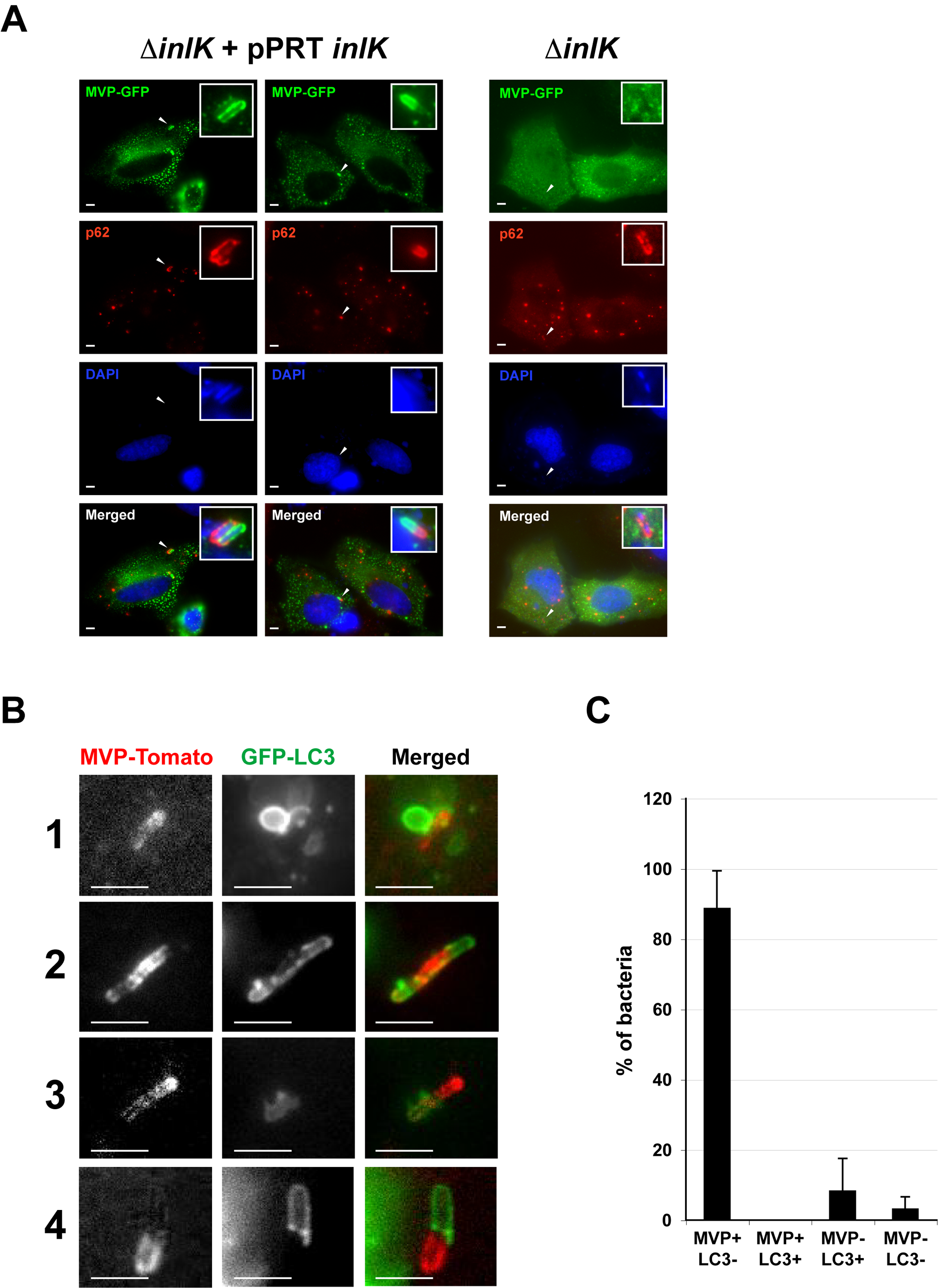

Supplement: Figure S4 — MVP and autophagy markers do not co-localize. A. Impaired recruitment of p62 to MVP positive Listeria. HeLa cells were transfected with MVP-GFP (green), infected with InlK over-expressing Listeria (ΔinlK+pPRT inlK) (left panel) or ΔinlK Listeria (right panel) for 4 h, fixed for fluorescence light microscopy, and stained with phalloidin (blue) and anti-p62 antibody (red). Inset regions are magnified. Arrows indicate independent bacteria The scale bar represents 1 µm. The vast majority of MVP-positive bacteria were completely devoid of anti-p62 labeling (95.1±2.0%; mean ± SEM from n = 3 experiments) but 4.9±2.0% (mean ± SEM from n = 3 experiments) were stained at one pole with MVP and at the other pole with p62. B. Polar recruitment of GFP-LC3 to MVP positive Listeria. HeLa cells were transfected with MVP-tomato (red) and GFP-LC3 (green), infected with InlK over-expressing Listeria (ΔinlK+pPRT inlK) for 4 h, fixed for fluorescence light microscopy. Four different bacteria are shown. The scale bar represents 1 µm. C. Quantification of MVP and LC3 recruitment at the surface of ΔactA overexpressing InlK (mean%±SEM%). Quantifications correspond to the data represented in Figure 5D. The percentages MVP+/LC3+ bacteria, MVP+/LC3-, MVP-/LC3+ and MVP-/LC3- were 88.6±12.8%, 0.0±0.0%, 8.2±9.3% and 3.1±3.5% respectively. Statistical analyses were performed on the results of 3 independent experiments using the Student's t test. (TIF) [file ppat.1002168.s004.tif]
