# Supplementary material for: Recruitment of the Major Vault Protein by InlK: A Listeria monocytogenes Strategy to Avoid Autophagy
Source: PLoS Pathog. 2011 Aug 4;7(8):e1002168. doi: 10.1371/journal.ppat.1002168 (PMC3150275; doi:10.1371/journal.ppat.1002168)
Supplement: Table S1 — L. monocytogenes strains used in this study. (DOC) [file ppat.1002168.s005.doc]

**Table S1 Recapitulative table of InlK prey obtained with the yeast two hybrid**

| Clone Number | Gene Name (Best Match) | PBS score | Start | Stop | Frame | Type Seq | % Id 5p/3p | Additional Gene Notes |
| --- | --- | --- | --- | --- | --- | --- | --- | --- |
| 1 | Homo sapiens - FAM46C | D | -43 | 853 | IF | 5p 3p | 96.3 / 92.5 | FAM46C; [gi|96975056|ref|NM_017709.3| Homo sapiens family with sequence similarity 46, member C (FAM46C), mRNA] |
| 2 | Homo sapiens - FAM46C | D | -43 | 853 | IF | 5p 3p | 94.3 / 94.2 | FAM46C; [gi|96975056|ref|NM_017709.3| Homo sapiens family with sequence similarity 46, member C (FAM46C), mRNA] |
| 3 | Homo sapiens - FBXO21 | D | 1464 | 2436 | IF | 5p 3p | 99.9 / 99.7 | FBXO21; [gi|53692180|ref|NM_033624.2| Homo sapiens F-box protein 21 (FBXO21), transcript variant 1, mRNA] |
| 4 | Homo sapiens - IL12RB2 | D | 465 | 937 | IF | 3p | 85.4 | IL12RB2; [gi|24497441|ref|NM_001559.2| Homo sapiens interleukin 12 receptor, beta 2 (IL12RB2), mRNA] |
| 5 | Homo sapiens - IL12RB2 | D | 465 | 941 | IF | 3p | 82.2 | IL12RB2; [gi|24497441|ref|NM_001559.2| Homo sapiens interleukin 12 receptor, beta 2 (IL12RB2), mRNA] |
| 6 | Homo sapiens - IL12RB2 | D | 465 | 936 | IF | 3p | 83.3 | IL12RB2; [gi|24497441|ref|NM_001559.2| Homo sapiens interleukin 12 receptor, beta 2 (IL12RB2), mRNA] |
| 7 | Homo sapiens - IL12RB2 | D | 465 | 936 | IF | 5p 3p | 94.3 / 98.7 | IL12RB2; [gi|24497441|ref|NM_001559.2| Homo sapiens interleukin 12 receptor, beta 2 (IL12RB2), mRNA] |
| 8 | Homo sapiens - IL12RB2 | D | 465 | 937 | IF | 5p | 86.2 | IL12RB2; [gi|24497441|ref|NM_001559.2| Homo sapiens interleukin 12 receptor, beta 2 (IL12RB2), mRNA] |
| 9 | Homo sapiens - IL12RB2 | D | 465 | 936 | IF | 5p 3p | 80.2 / 89.0 | IL12RB2; [gi|24497441|ref|NM_001559.2| Homo sapiens interleukin 12 receptor, beta 2 (IL12RB2), mRNA] |
| 10 | Homo sapiens - IL12RB2 | D | 465 | 939 | IF | 5p | 83.8 | IL12RB2; [gi|24497441|ref|NM_001559.2| Homo sapiens interleukin 12 receptor, beta 2 (IL12RB2), mRNA] |
| 11 | Homo sapiens - IL12RB2 | D | 465 | 936 | IF | 5p | 85.1 | IL12RB2; [gi|24497441|ref|NM_001559.2| Homo sapiens interleukin 12 receptor, beta 2 (IL12RB2), mRNA] |
| 12 | Homo sapiens - IL12RB2 | D | 465 | 936 | IF | 5p | 87.5 | IL12RB2; [gi|24497441|ref|NM_001559.2| Homo sapiens interleukin 12 receptor, beta 2 (IL12RB2), mRNA] |
| 13 | Homo sapiens - IL12RB2 | D | 465 | 936 | IF | 5p 3p | 90.0 / 94.9 | IL12RB2; [gi|24497441|ref|NM_001559.2| Homo sapiens interleukin 12 receptor, beta 2 (IL12RB2), mRNA] |
| 14 | Homo sapiens - IL12RB2 | D | 465 | 936 | IF | 5p | 99.6 | IL12RB2; [gi|24497441|ref|NM_001559.2| Homo sapiens interleukin 12 receptor, beta 2 (IL12RB2), mRNA] |
| 15 | Homo sapiens - IL12RB2 | D | 465 | 936 | IF | 5p 3p | 83.8 / 97.5 | IL12RB2; [gi|24497441|ref|NM_001559.2| Homo sapiens interleukin 12 receptor, beta 2 (IL12RB2), mRNA] |
| 16 | Homo sapiens - IL12RB2 | D | 465 | 936 | IF | 5p 3p | 73.4 / 93.2 | IL12RB2; [gi|24497441|ref|NM_001559.2| Homo sapiens interleukin 12 receptor, beta 2 (IL12RB2), mRNA] |
| 17 | Homo sapiens - IL12RB2 | D | 465 | 938 | IF | 5p | 84.1 | IL12RB2; [gi|24497441|ref|NM_001559.2| Homo sapiens interleukin 12 receptor, beta 2 (IL12RB2), mRNA] |
| 18 | Homo sapiens - JUND | D | 696 | 1566 | IF | 5p 3p | 90.3 / 91.5 | JUND; [gi|169234622|ref|NM_005354.4| Homo sapiens jun D proto-oncogene (JUND), mRNA] |
| 19 | Homo sapiens - LOC51058 | D | 315 | 1465 | IF | 5p 3p | 95.5 / 98.1 | LOC51058; [gi|24475961|ref|NM_015911.2| Homo sapiens hypothetical protein LOC51058 (LOC51058), mRNA] |
| 20 | Homo sapiens - LOC51058 | D | 315 | 1465 | IF | 5p 3p | 95.9 / 93.2 | LOC51058; [gi|24475961|ref|NM_015911.2| Homo sapiens hypothetical protein LOC51058 (LOC51058), mRNA] |
| 21 | Homo sapiens - LOC51058 | D | 315 | 1465 | IF | 5p 3p | 98.1 / 98.5 | LOC51058; [gi|24475961|ref|NM_015911.2| Homo sapiens hypothetical protein LOC51058 (LOC51058), mRNA] |
| 22 | Homo sapiens - LOC51058 | D | 315 | 1465 | IF | 5p 3p | 95.9 / 98.3 | LOC51058; [gi|24475961|ref|NM_015911.2| Homo sapiens hypothetical protein LOC51058 (LOC51058), mRNA] |
| 23 | Homo sapiens - MVP | A | No Data | 1080 | ?? | 3p | 99.2 | MVP; [gi|19913411|ref|NM_005115.3| Homo sapiens major vault protein (MVP) transcript variant 2, mRNA] |
| 24 | Homo sapiens - MVP | A | No Data | 1154 | ?? | 3p | 90.0 | MVP; [gi|19913411|ref|NM_005115.3| Homo sapiens major vault protein (MVP) transcript variant 2, mRNA] |
| 25 | Homo sapiens - MVP | A | No Data | 1149 | ?? | 3p | 93.5 | MVP; [gi|19913411|ref|NM_005115.3| Homo sapiens major vault protein (MVP) transcript variant 2, mRNA] |
| 26 | Homo sapiens - MVP | A | 39 | 1081 | IF | 5p 3p | 91.2 / 94.1 | MVP; [gi|19913411|ref|NM_005115.3| Homo sapiens major vault protein (MVP) transcript variant 2, mRNA] |
| 27 | Homo sapiens - MVP | A | 39 | 1081 | IF | 5p 3p | 92.0 / 98.6 | MVP; [gi|19913411|ref|NM_005115.3| Homo sapiens major vault protein (MVP) transcript variant 2, mRNA] |
| 28 | Homo sapiens - MVP | A | 45 | 915 | IF | 5p 3p | 98.2 / 96.7 | MVP; [gi|19913411|ref|NM_005115.3| Homo sapiens major vault protein (MVP) transcript variant 2, mRNA] |
| 29 | Homo sapiens - MVP | A | 63 | 1150 | IF | 5p 3p | 92.0 / 91.2 | MVP; [gi|19913411|ref|NM_005115.3| Homo sapiens major vault protein (MVP) transcript variant 2, mRNA] |
| 30 | Homo sapiens - MVP | A | 114 | 1173 | IF | 5p 3p | 95.6 / 99.2 | MVP; [gi|19913411|ref|NM_005115.3| Homo sapiens major vault protein (MVP) transcript variant 2, mRNA] |
| 31 | Homo sapiens - MVP | A | 153 | 1576 | IF | 5p 3p | 98.8 / 97.0 | MVP; [gi|19913411|ref|NM_005115.3| Homo sapiens major vault protein (MVP) transcript variant 2, mRNA] |
| 32 | Homo sapiens - MVP | A | 153 | 1576 | IF | 5p 3p | 93.3 / 98.2 | MVP; [gi|19913411|ref|NM_005115.3| Homo sapiens major vault protein (MVP) transcript variant 2, mRNA] |
| 33 | Homo sapiens - MVP | A | 153 | 1576 | IF | 5p 3p | 97.8 / 95.8 | MVP; [gi|19913411|ref|NM_005115.3| Homo sapiens major vault protein (MVP) transcript variant 2, mRNA] |
| 34 | Homo sapiens - MVP | A | 153 | 1576 | IF | 5p 3p | 98.7 / 97.0 | MVP; [gi|19913411|ref|NM_005115.3| Homo sapiens major vault protein (MVP) transcript variant 2, mRNA] |
| 35 | Homo sapiens - MVP | A | 153 | 1576 | IF | 5p 3p | 94.9 / 98.0 | MVP; [gi|19913411|ref|NM_005115.3| Homo sapiens major vault protein (MVP) transcript variant 2, mRNA] |
| 36 | Homo sapiens - MVP | A | 408 | 1007 | IF | 5p 3p | 95.8 / 91.8 | MVP; [gi|19913411|ref|NM_005115.3| Homo sapiens major vault protein (MVP) transcript variant 2, mRNA] |
| 37 | Homo sapiens - MVP | A | 408 | 1007 | IF | 5p 3p | 93.0 / 96.5 | MVP; [gi|19913411|ref|NM_005115.3| Homo sapiens major vault protein (MVP) transcript variant 2, mRNA] |
| 38 | Homo sapiens - MVP | A | 480 | 1325 | IF | 5p 3p | 97.5 / 96.5 | MVP; [gi|19913411|ref|NM_005115.3| Homo sapiens major vault protein (MVP) transcript variant 2, mRNA] |
| 39 | Homo sapiens - MVP | A | 480 | 1325 | IF | 5p 3p | 94.3 / 95.8 | MVP; [gi|19913411|ref|NM_005115.3| Homo sapiens major vault protein (MVP) transcript variant 2, mRNA] |
| 40 | Homo sapiens - MVP | A | 480 | 1325 | IF | 5p 3p | 89.6 / 94.7 | MVP; [gi|19913411|ref|NM_005115.3| Homo sapiens major vault protein (MVP) transcript variant 2, mRNA] |
| 41 | Homo sapiens - MVP | A | 480 | 1325 | IF | 5p 3p | 96.2 / 96.8 | MVP; [gi|19913411|ref|NM_005115.3| Homo sapiens major vault protein (MVP) transcript variant 2, mRNA] |
| 42 | Homo sapiens - MVP | A | 540 | 1291 | IF | 5p 3p | 99.4 / 95.2 | MVP; [gi|19913411|ref|NM_005115.3| Homo sapiens major vault protein (MVP) transcript variant 2, mRNA] |
| 43 | Homo sapiens - MVP | A | 546 | 1155 | IF | 5p 3p | 93.8 / 90.0 | MVP; [gi|19913411|ref|NM_005115.3| Homo sapiens major vault protein (MVP) transcript variant 2, mRNA] |
| 44 | Homo sapiens - MVP | A | 546 | 1155 | IF | 5p 3p | 94.4 / 91.0 | MVP; [gi|19913411|ref|NM_005115.3| Homo sapiens major vault protein (MVP) transcript variant 2, mRNA] |
| 45 | Homo sapiens - MVP | A | 546 | 1155 | IF | 5p 3p | 96.6 / 96.1 | MVP; [gi|19913411|ref|NM_005115.3| Homo sapiens major vault protein (MVP) transcript variant 2, mRNA] |
| 46 | Homo sapiens - MVP | A | 546 | 1155 | IF | 5p 3p | 94.8 / 94.6 | MVP; [gi|19913411|ref|NM_005115.3| Homo sapiens major vault protein (MVP) transcript variant 2, mRNA] |
| 47 | Homo sapiens - MVP | A | 546 | 1155 | IF | 5p 3p | 94.8 / 97.2 | MVP; [gi|19913411|ref|NM_005115.3| Homo sapiens major vault protein (MVP) transcript variant 2, mRNA] |
| 48 | Homo sapiens - MVP | A | 546 | 1155 | IF | 5p 3p | 95.5 / 95.3 | MVP; [gi|19913411|ref|NM_005115.3| Homo sapiens major vault protein (MVP) transcript variant 2, mRNA] |
| 49 | Homo sapiens - MVP | A | 546 | 1155 | IF | 5p 3p | 99.0 / 97.8 | MVP; [gi|19913411|ref|NM_005115.3| Homo sapiens major vault protein (MVP) transcript variant 2, mRNA] |
| 50 | Homo sapiens - MVP | A | 546 | No Data | IF | 5p | 92.8 | MVP; [gi|19913411|ref|NM_005115.3| Homo sapiens major vault protein (MVP) transcript variant 2, mRNA] |
| 51 | Homo sapiens - MVP | A | 567 | 2012 | IF | 5p 3p | 99.4 / 99.5 | MVP; [gi|19913411|ref|NM_005115.3| Homo sapiens major vault protein (MVP) transcript variant 2, mRNA] |
| 52 | Homo sapiens - MVP | A | 597 | 1666 | IF | 5p 3p | 95.7 / 96.0 | MVP; [gi|19913411|ref|NM_005115.3| Homo sapiens major vault protein (MVP) transcript variant 2, mRNA] |
| 53 | Homo sapiens - MVP | A | 597 | 1666 | IF | 5p 3p | 92.7 / 94.7 | MVP; [gi|19913411|ref|NM_005115.3| Homo sapiens major vault protein (MVP) transcript variant 2, mRNA] |
| 54 | Homo sapiens - MVP | A | 609 | 1135 | IF | 5p 3p | 92.8 / 95.5 | MVP; [gi|19913411|ref|NM_005115.3| Homo sapiens major vault protein (MVP) transcript variant 2, mRNA] |
| 55 | Homo sapiens - MVP | A | 609 | 1134 | IF | 3p | 98.5 | MVP; [gi|19913411|ref|NM_005115.3| Homo sapiens major vault protein (MVP) transcript variant 2, mRNA] |
| 56 | Homo sapiens - MVP | A | 612 | 1162 | IF | 5p 3p | 95.1 / 88.4 | MVP; [gi|19913411|ref|NM_005115.3| Homo sapiens major vault protein (MVP) transcript variant 2, mRNA] |
| 57 | Homo sapiens - MVP | A | 624 | 1136 | IF | 5p 3p | 96.7 / 96.9 | MVP; [gi|19913411|ref|NM_005115.3| Homo sapiens major vault protein (MVP) transcript variant 2, mRNA] |
| 58 | Homo sapiens - MVP | A | 624 | 1136 | IF | 5p 3p | 92.4 / 97.5 | MVP; [gi|19913411|ref|NM_005115.3| Homo sapiens major vault protein (MVP) transcript variant 2, mRNA] |
| 59 | Homo sapiens - MVP | A | 624 | 1136 | IF | 5p 3p | 96.1 / 97.5 | MVP; [gi|19913411|ref|NM_005115.3| Homo sapiens major vault protein (MVP) transcript variant 2, mRNA] |
| 60 | Homo sapiens - MVP | A | 624 | 1136 | IF | 5p 3p | 90.4 / 98.6 | MVP; [gi|19913411|ref|NM_005115.3| Homo sapiens major vault protein (MVP) transcript variant 2, mRNA] |
| 61 | Homo sapiens - MVP | A | 624 | 1136 | IF | 5p 3p | 95.9 / 98.3 | MVP; [gi|19913411|ref|NM_005115.3| Homo sapiens major vault protein (MVP) transcript variant 2, mRNA] |
| 62 | Homo sapiens - MVP | A | 624 | 1136 | IF | 5p 3p | 99.8 / 91.4 | MVP; [gi|19913411|ref|NM_005115.3| Homo sapiens major vault protein (MVP) transcript variant 2, mRNA] |
| 63 | Homo sapiens - MVP | A | 624 | 1136 | IF | 5p 3p | 99.6 / 99.4 | MVP; [gi|19913411|ref|NM_005115.3| Homo sapiens major vault protein (MVP) transcript variant 2, mRNA] |
| 64 | Homo sapiens - MVP | A | 624 | 1514 | IF | 5p 3p | 96.5 / 98.2 | MVP; [gi|19913411|ref|NM_005115.3| Homo sapiens major vault protein (MVP) transcript variant 2, mRNA] |
| 65 | Homo sapiens - MVP | A | 624 | 1514 | IF | 5p 3p | 97.4 / 98.0 | MVP; [gi|19913411|ref|NM_005115.3| Homo sapiens major vault protein (MVP) transcript variant 2, mRNA] |
| 66 | Homo sapiens - GenMatch | D | -1 | 940 | IF | 5p 3p | 95.1 / 94.6 | unknown; [prey946684 - Human - GenMatch] |
| 67 | Homo sapiens - GenMatch | D | -1 | 940 | IF | 5p 3p | 93.7 / 95.2 | unknown; [prey946684 - Human - GenMatch] |
| 68 | Homo sapiens - GenMatch | D | -1 | 940 | IF | 5p 3p | 92.2 / 96.7 | unknown; [prey946684 - Human - GenMatch] |
| 69 | Homo sapiens - GenMatch | D | -1 | 940 | IF | 5p 3p | 94.1 / 94.4 | unknown; [prey946684 - Human - GenMatch] |
| **Summary of PBS categories** | |  |  |  |  |  |  |  |
| A : Very high confidence in the interaction | | | |  |  |  |  |  |
| B : High confidence in the interaction | | |  |  |  |  |  |  |
| C : Good confidence in the interaction | | |  |  |  |  |  |  |
| D : Moderate confidence in the interaction | | | |  |  |  |  |  |
| This category is the most difficult to interpret because it mixes two classes of interactions : | | | | | | | | |
| * False-positive interactions | |  |  |  |  |  |  |  |
| * Interactions hardly detectable by the Y2H technique (low representation of the mRNA in the library, prey folding, prey toxicity in yeast...) | | | | | | | | |
| E : Interactions involving highly connected prey domains, warning of non-specific interaction. The threshold for high connectivity is 10 for | | | | | | | | |
| screens with Human, Mouse, Drosophila and Arabidopsis and 6 for all other organisms. They can be classified in different categories: | | | | | | | | |
| * Prey proteins that are known to be highly connected due to their biological function | | | | | | | |  |
| * Proteins with a prey interacting domain that contains a known protein interaction motif or a biochemically promiscuous motif | | | | | | | | |
| F : Experimentally proven technical artifacts | | | |  |  |  |  |  |
| N/A : The PBS is a score that is automatically computed through algorithms and cannot be attributed for the following reasons : | | | | | | | | |
| * All the fragments of the same reference CDS are antisens | | | | | |  |  |  |
| * The 5p sequence is missing | |  |  |  |  |  |  |  |
| * All the fragments of the same reference CDS are either all OOF1 or all OOF2 | | | | | | | |  |
| * All the fragments of the same reference CDS lie in the 5' or 3' UTR | | | | | | |  |  |
