# Supplementary material for: Recruitment of the Major Vault Protein by InlK: A Listeria monocytogenes Strategy to Avoid Autophagy
Source: PLoS Pathog. 2011 Aug 4;7(8):e1002168. doi: 10.1371/journal.ppat.1002168 (PMC3150275; doi:10.1371/journal.ppat.1002168)
Supplement: Table S2 — Results of the yeast two-hybrid screening. The L. monocytogenes wild-type [72] and the ΔactA [73] were previously published. (DOC) [file ppat.1002168.s006.doc]

**Table S2. *L. monocytogenes* strains used in this study.**

| **Strain** | **Description** | **Collection number** | **Antibiotic selection** | **Source** |
| --- | --- | --- | --- | --- |
| EGD-e | *L. monocytogenes* EGD-e WT strain | BUG 1600 | no | [39] |
| ∆*inlK* | *L. monocytogenes* EGD-e deleted in the *inlK* encoding gene | BUG 2166 | no | Present study |
| ∆*inlK*-(pPL2-*inlK*) | EGD-e∆*inlK* complemented with an inserted chromosomal monocopy of *inlK* gene preceded by its own promoter | BUG 2813 | no or Cm | Present study |
| ∆*inlK*-(pPRT-*inlK*) | EGD-e∆*inlK* + (pPRT-*inlK*) | BUG 2814 | Ery | Present study |
| ∆*inlK*-(pPRT-empty) | EGD-e∆*inlK* + (pPRT-empty) | BUG 2956 | Ery | Present study |
| ∆*srtA*-(pPRT-*inlK*) | EGD-e∆*srtA* + (pPRT-*inlK*) | BUG 2815 | Ery | Present study |
| EGD-e-(pPRT-empty) | EGD-e + (pPRT-empty) | BUG 2293 | Ery | [49] |
| EGD-e-(pPRT-*inlJ*) | EGD-e + (pPRT-*inlJ*) | BUG 2350 | Ery | [49] |
| EGD | *L. monocytogenes* EGD WT strain | BUG 600 | no | [72] |
| EGD-(pADc-*InlK*) | EGD + (pADc-*inlK*) | BUG 3007 | no or Cm | Present study |
| EGD-(pADc-GFP) | EGD + (pADc-*gfp*) | BUG 2539 | no or Cm | [48] |
| D*actA* | EGD ∆*actA* | BUG 2140 | no | [73] |
| ∆*actA*-(pADc-GFP) | EGD ∆*actA* + (pADc*-gfp*) | BUG 2783 | no or Cm | [48] |
| ∆*actA*-(pADc-*inlK*) | EGD ∆*actA* + (pADc*-inlK*) | BUG 2973 | no or Cm | Present study |
| EGD-e-(pPL2-P*_hly_*-*lux*_ABCDE_) | EGD-e + (pPL2-P*_hly_*-*lux*_ABCDE_) | BUG 3075 | No or Cm | Present study |
| EGD-e-(pPL2-P*_inlK_*-*lux*_ABCDE_) | EGD-e + (pPL2-P*_inlK_*-*lux*_ABCDE_) | BUG 2816 | No or Cm | Present study |

Cm = Chloramphenicol, Ery = Erythromycin
